# TRAINSPOTTER: profiling nascent protein N-termini indicative of bacterial translation initiation via deformylation-assisted N-terminomics

**DOI:** 10.1093/nar/gkag587

**Published:** 2026-06-08

**Authors:** Petra Van Damme

**Affiliations:** iRIP Unit, Laboratory of Microbiology, Department of Biochemistry and Microbiology, Faculty of Sciences, Ghent University, Ghent 9000, Belgium

## Abstract

Accurate delineation of bacterial translation initiation sites (TISs) remains a major challenge, as conventional genome annotation and ribosome profiling (Ribo-seq) often lack the resolution to discriminate closely spaced start codons. To overcome these limitations, we developed TRAINSPOTTER (TRAnslation INitiation SPOTTER), a deformylation-assisted N-terminomics workflow that enables direct, proteome-wide detection of nascent N-termini indicative of active translation initiation. TRAINSPOTTER exploits the universal N-terminal formylation of initiator methionine in bacteria: *in vitro* enzymatic deformylation by peptide deformylase (PDF) generates a diagnostic hydrophilic shift, allowing selective isolation of previously formylated, initiation-derived peptides by COFRADIC-based chromatography. Optional *in vivo* PDF inhibition transiently enriches formylated N-termini, primarily enhancing detection sensitivity. Integration of pulse SILAC (Stable Isotope Labeling by Amino acids in Cell Culture) labeling confirmed that deformylation-shifted peptides represent newly synthesized N-termini, validating TRAINSPOTTER’s specificity for nascent translation products. Application to *Escherichia coli* enabled precise mapping of >1000 TIS-indicative N-termini, including numerous alternative and near-cognate start sites, providing direct proteomic evidence for co-expressed N-terminal proteoforms. The method complements and refines Ribo-seq datasets, offering amino acid-level resolution for otherwise ambiguous initiation events. TRAINSPOTTER thus establishes a robust biochemical framework for proteome-wide identification of TISs and advances the experimental annotation of bacterial proteomes.

## Graphical Abstract

**Graphical Abstract:**
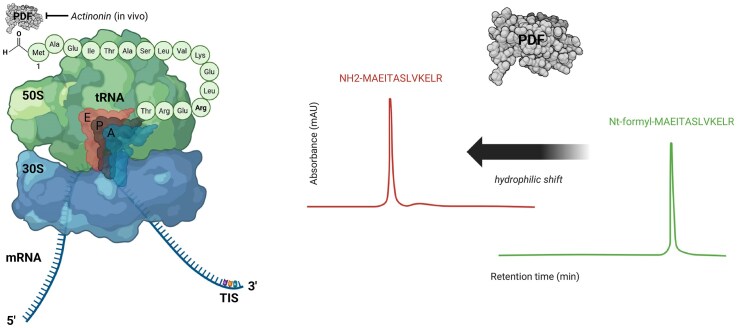

## Introduction

**Figure 1.**
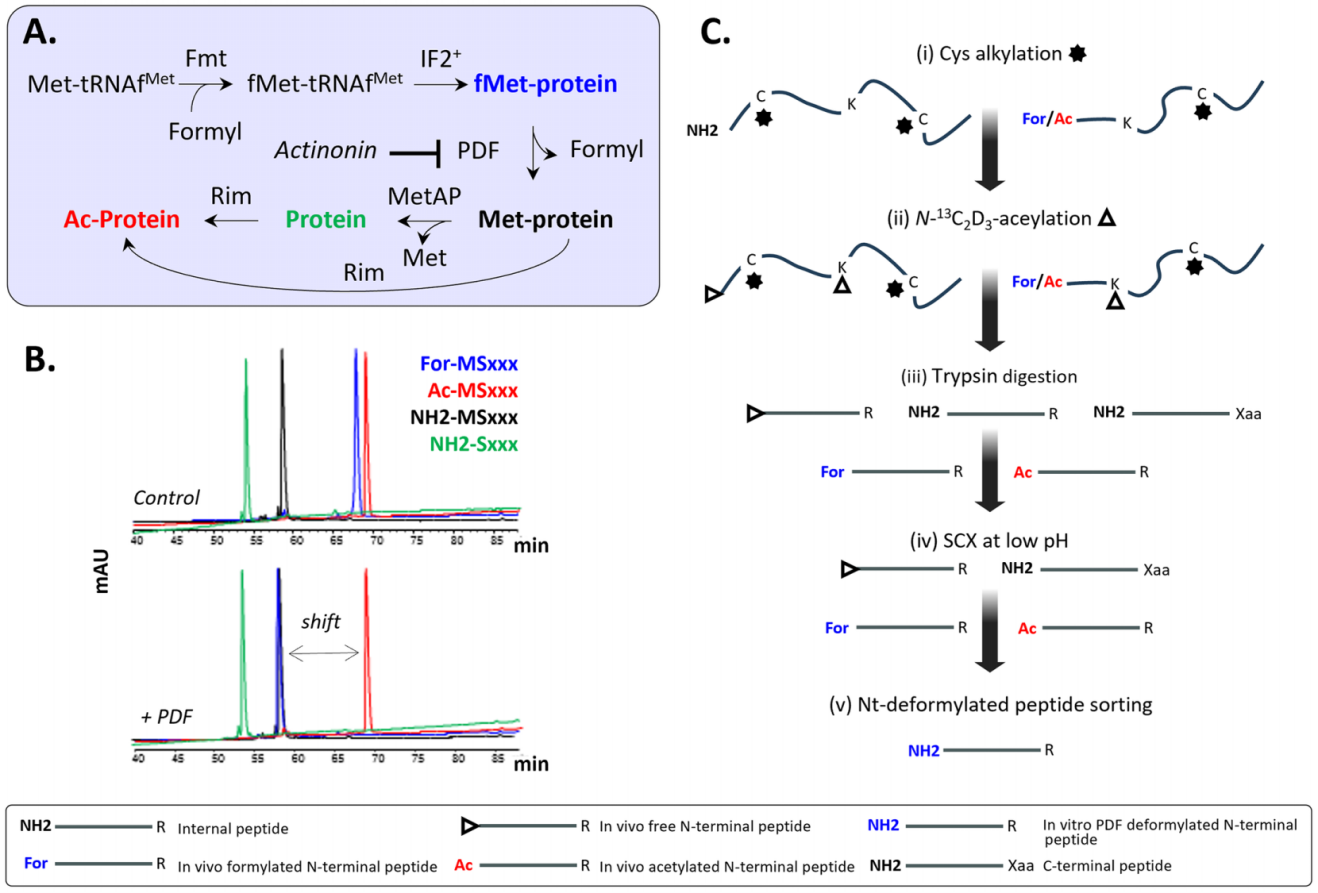
Peptide deformylase (PDF)-mediated deformylation induces a diagnostic hydrophilic shift, enabling chromatographic isolation of initiation-derived N-termini in TRAINSPOTTER. (**A**) Overview of (co-translational) N-terminal processing in bacteria. Translation initiates with fMet delivered by Met-tRNA^fMet^, generated through formylation by Fmt. PDF co-translationally removes the formyl group, after which methionine aminopeptidase (MetAP) may excise the initiator Met (iMet). In bacteria, N-terminal acetylation is restricted to a limited subset of proteins, occurs typically at low stoichiometry and is catalyzed by Rim N-terminal acetyltransferases either on the iMet or on the MetAP-processed N-terminus [6, 52]. actinonin inhibits PDF activity, stabilizing Nt-formyl groups *in vivo*. IF2^+^ denotes the active, GTP-bound form of initiation factor 2. (**B**) Reversed-phase (RP)-HPLC (high-performance liquid chromatography) analysis demonstrating the specificity of PDF for Nt-formyl peptides. Overlaid chromatograms (0.1% TFA, pH 3.0) of synthetic peptides show that only the Nt-formylated peptide (blue) undergoes a pronounced hydrophilic retention-time shift upon PDF treatment, whereas Nt-acetylated (red), Nt-free (black), and iMet-removed Nt-free (green) variants retain their original retention times. UV absorbance at 214 nm (mAU) is shown on the *Y*-axis. (**C**) Schematic overview of the TRAINSPOTTER workflow. Proteins are (i) alkylated, (ii) N-terminally blocked *in vitro* by *N*-^13^C_2_D_3_-acetylation (^13^C_2_D_3_-Ac or heavy Ac), and (iii) digested with trypsin. After (iv) strong cation exchange (SCX) separation at low pH, the SCX flow-through is subjected to primary RP-HPLC fractionation. Each primary fraction is incubated with recombinant PDF prior to secondary RP-HPLC re-separation, converting formylated N-termini into free NH_2_ N-termini and inducing a characteristic hydrophilic shift. This deformylation-dependent shift defines the specificity of the workflow and enables COFRADIC-based sorting of Nt-deformylated (shifted/off-diagonal) peptides from blocked (diagonal) N-terminal and C-terminal peptides, whereas *in vivo* PDF inhibition can be used to enhance detection sensitivity by increasing the abundance of Nt-formylated substrates. Symbols and colors denote internal peptides (NH_2_; black), *in vivo* free N-terminal peptides (triangle), *in vivo* formylated N-terminal peptides (For), *in vivo* acetylated N-terminal peptides (Ac), *in vitro* PDF-deformylated N-terminal peptides (NH_2_; blue), and C-terminal peptides (NH_2_-Xaa).

Bacterial protein synthesis universally initiates with N-formylmethionine (fMet) [1], delivered by a specialized initiator transfer RNA (fMet-tRNA). This is a defining characteristic of prokaryotic translation, distinguishing it from non-organelle-specific eukaryotic translation initiation, which typically employs unformylated methionine. The formyl moiety is enzymatically added to Met-tRNA by methionyl-tRNA formyltransferase (Fmt), encoded by *fmt*. Although Fmt plays a role in enhancing translation initiation efficiency, genetic studies have shown that it is generally non-essential for bacterial viability [2, 3], suggesting compensatory mechanisms can bypass the formylation requirement (Fig. 1A).

Immediately after initiation, as the nascent peptide emerges from the ribosomal exit tunnel, the N-terminal formyl group is typically removed co-translationally by PDF [4, 5]. This is a critical and highly prevalent processing step in bacterial protein maturation, affecting over 95% of all bacterial proteins [6] and is considered essential in most bacteria, as deletion of *def* (encoding PDF) is lethal [7] (Fig. 1A). Remarkably, this lethality can be suppressed by simultaneous deletion of *fmt* [8, 9], implying that the toxicity or cellular burden arises from retained formyl groups rather than the absence of deformylation per se. Consistent with this, the small-molecule antibiotic actinonin—a potent and highly specific inhibitor of PDF—has been widely used to probe the physiological consequences of deformylation blockade in bacteria [6, 10]. Following deformylation, MetAP frequently removes the iMet, affecting the majority of bacterial N-termini [11]. MetAP acts sequentially to PDF and is universally conserved [12] (Fig. 1A).

Despite their ubiquity, the precise physiological roles underlying the essentiality of PDF and MetAP remain debated. Proposed functions include efficient methionine recycling [13], specialized roles for iMet-processed N-termini [14], and regulation of protein stability via the bacterial fMet/N-degron pathway, where formylated N-termini act as degradation signals [15].

The advent of next-generation sequencing technologies, ribosome profiling (Ribo-seq) in particular, has revolutionized the study of translation [16, 17]. Ribo-seq provides genome-wide snapshots of actively translating ribosomes, offering unprecedented insights into translational control and leading to the discovery of numerous previously unannotated translated open reading frames (ORFs), more appropriately described as translons [18], including small translated ORFs (sORFs; small translons) and alternative translons [16, 17, 19–21]. It has become increasingly clear that protein synthesis is a key contributor to protein abundance [22, 23–24]. This emphasizes the critical importance of directly monitoring translational changes at the proteome level, distinguishing them from protein degradation, to accurately understand overall steady-state protein levels and translational control mechanisms.

Despite its transformative power, Ribo-seq exhibits intrinsic limitations for precisely delineation of translation initiation sites (TISs) in bacteria [19, 25–27]. Closely spaced initiation codons are difficult to discriminate, as positional uncertainty in ribosome footprint assignment—further compounded by footprint-length variability and Shine–Dalgarno-associated effects—limits the unambiguous attribution of initiation events to individual start sites [19, 25–28]. As a consequence, ambiguities frequently arise for alternative or nearby initiation events, where ribosome-footprint density alone cannot reliably assign the exact start codon. This inherent resolution limitation can lead to inaccuracies in genome annotation, further aggravated by the tendency of *in silico* prediction algorithms to default to the longest possible ORF, thereby overlooking genuine alternative translons, each initiated from distinct TISs [29].

In addition, although a three-nucleotide (nt) periodicity (triplet periodicity) is often detectable in bacterial Ribo-seq when data are aggregated across many genes (i.e. in metagene analyses), in practice, this periodicity is frequently weak, variable, or ambiguous in bacteria at the level of individual genes [30], limiting its utility for precise translon boundary delineation or TIS assignment. Finally, Ribo-seq-based TIS detection commonly relies on peak calling and thresholding relative to local background; however, peak height can be influenced by factors unrelated to initiation efficiency, including sequence- and library-preparation biases and drug-induced ribosome stalling. Beyond algorithmic thresholds, initiation-inhibitor datasets can also reflect compound-specific stalling behaviors, and more generally, inhibitor-based initiation mapping can generate apparent start-like peaks that require cautious interpretation and orthogonal validation [31].

Altogether, these limitations highlight the critical need for complementary, orthogonal approaches that provide direct biochemical evidence for translation initiation events and enable high-resolution mapping of TISs.

In this context, TRAINSPOTTER (TRAnslation INitiation SPOTTER) contributes such evidence by exploiting protein N-termini—particularly those retaining the initiator fMet—as direct biochemical proxies of translation initiation, thereby enabling sequence-predicted ORFs to be experimentally resolved into bona fide translons. Identifying (nascent) N-terminal peptides can therefore deliver unambiguous, peptide-level evidence for both canonical and alternative initiation events. This complementarity establishes the foundation for riboproteogenomic integration, where Ribo-seq offers global translational context and N-terminomics provides molecular confirmation of initiation sites [29, 32, 33].

N-terminomics, a proteomic strategy involving the selective enrichment of protein N-terminal peptides prior to liquid chromatography-tandem mass spectrometry (LC-MS/MS) analysis, is invaluable for mapping protein start sites and N-terminal processing events [34]. These N-termini, and the iMet and its modifications in particular, serve as direct proxies of translation initiation and subsequent protein processing. Our prior work developed and refined the N-terminal COFRADIC (COmbined FRActional DIagonal Chromatography) methodology [35–37]. Coupled with proteome labeling [e.g. SILAC (Stable Isotope Labeling by Amino acids in Cell Culture)] and amine-reactive mass tagging, this N-terminomics approach has proven to be a powerful tool for studying translation dynamics [38].

While previous N-terminal COFRADIC implementations [1] and related approaches such as LATE (LysN amino terminal enrichment) [39] and TAILS (terminal amine isotopic labeling of substrates) [40] have successfully enriched N-terminal peptides using a variety of chemical derivatizations and chromatographic strategies, these approaches were not specifically designed to distinguish nascent, initiation-derived N-termini from other N-terminal species at the proteome-wide level, nor to exploit the unique N-terminal formylation that characterizes bacterial translation initiation.

Early work by Bienvenut *et al*. [6] provided the first proteome-wide quantitative characterization of bacterial N-terminal modification states and demonstrated that global N-termini enrichment combined with PDF inhibition increases the representation of Nt-formylated N-termini in *Escherichia coli* Δ*tolC* backgrounds. A COFRADIC-based proteogenomic application in *Listeria monocytogenes* similarly illustrated the translational potential of N-terminomics by leveraging PDF inhibition to enrich initiating N-termini [41].

Together, these studies established the feasibility of detecting formylated N-termini at scale and highlighted the impact of PDF inhibition on Nt-formyl retention.

Building on these principles, we developed a deformylation-assisted N-terminal COFRADIC approach, termed TRAINSPOTTER, that selectively targets the fMet modification characteristic of bacterial translation initiation. By combining controlled enzymatic PDF-mediated deformylation with chromatographic separation, TRAINSPOTTER introduces a distinct hydrophilic shift that differentiates previously formylated N-termini from all other peptides.

In contrast to existing N-terminomics workflows that rely on global enrichment of protein N-termini, TRAINSPOTTER uniquely combines N-terminal enrichment with a deformylation-dependent positive selection step, enabling the selective capture of previously Nt-formylated, initiation-derived N-termini. This biochemical specificity provides a direct and powerful proteome-wide readout of bacterial translation initiation at single-amino-acid resolution.

As demonstrated in the present study, TRAINSPOTTER enables proteome-wide mapping of bacterial translation initiation events by capturing deformylation-derived N-terminal peptides that reflect the transient formylated state of nascent proteins. Application of this workflow reveals both canonical and alternative TISs (aTISs) and uncovers previously unannotated N-terminal-proteoforms, thereby complementing and refining current riboproteogenomic annotations.

## Materials and methods

### *In vitro* deformylation of synthetic peptides by recombinant PDF

Recombinant *E. coli* PDF (19.4 kDa) (kindly provided by Profs Carmela Giglione and Thierry Meinnel) was expressed and purified as described previously [42]. Purified PDF stock solutions (459 µM; 8.9 mg/ml) were stored at 4°C in 50 mM HEPES (pH 7.5), 5 mM NiCl_2_, and 50% glycerol, conditions under which the enzyme remains stable for months without detectable loss of activity. Synthetic oligopeptides representing distinct N-terminal variants—Nt-free, Nt-formylated, Nt-acetylated, and Nt-free/iMet-processed—were synthesized by Fmoc solid-phase chemistry (Applied Biosystems 433A Peptide Synthesizer). Sequences were derived from elongation factor Ts [(M)AEITASLVKELR; UniProt P0A6P1] and enolase [(M)SKIVKVIGR; UniProt P64077]. To generate methionine sulfoxide-containing peptides, 100 nmol of Nt-formylated peptides (For-MAEITASLVKELR and For-MSKIVKVIGR) were incubated in 80 µl of 0.1% trifluoroacetic acid (TFA) with 20 µl of 3% (w/w) H_2_O_2_ for 30 min at 30°C. Following oxidation, residual H_2_O_2_ was removed by solid-phase extraction using AccuBONDII ODS-C18 cartridges (100 mg, Agilent). Cartridges were conditioned with 2 ml of 50% acetonitrile, equilibrated with 5 ml solvent A (0.1% TFA, 2% acetonitrile), washed with 5 ml of 2% acetonitrile, and peptides were eluted with 3 ml of 70% acetonitrile. Eluates were aliquoted and vacuum-dried. For deformylation assays, 20 nmol of peptide was dissolved in 100 µl of reaction buffer (50 mM HEPES, pH 7.5, 5 mM NiCl_2_) to a final concentration of 200 µM. Recombinant PDF was added to 1 µM, and reactions were incubated at 37°C. Samples were collected at 15, 30, 60, and 120 min. At each time point, 20 µl (equivalent to 4 nmol peptide) was withdrawn and quenched with 180 µl of 0.1% TFA. From this mixture, 50 µl (≈1 nmol) was injected onto the RP-HPLC column. Deformylation was monitored by RP-HPLC with UV detection at 214 nm, based on the appearance of product peaks and the corresponding reduction of precursor peptide peaks. The extent of deformylation was quantified from integrated peak surface areas. Peak annotation and confirmation of deformylation were performed by matrix-assisted laser desorption/ionization time-of-flight (MALDI-TOF) mass spectrometry (Bruker) and LC–MS/MS on an ESI-Q-TOF Premier instrument (Waters Corporation).

### Bacterial culture, actinonin treatment, and TRAINSPOTTER sample collection

The *E. coli* CAG12184 TolC-deficient strain (CGSC7437; F-, λ-, *tolC210*::Tn10, *rph-1*) was obtained from the *E. coli* Genetic Stock Collection [43]. To resuscitate the strain, a sterile filter disk impregnated with a small amount of glycerolized culture was placed onto an antibiotic-free Luria–Bertani (LB) agar plate (Miller’s formulation: 10 g/l Bacto peptone, 5 g/l Bacto yeast extract, 10 g/l NaCl, and 12 g/l agar). The disk was rehydrated with a drop of sterile LB broth (10 g/l Bacto peptone, 5 g/l Bacto yeast extract, 10 g/l NaCl) and the resulting suspension was streaked using glass beads to obtain isolated colonies. For routine culture, *E. coli* CAG12184 cells were grown overnight in 10 ml of LB medium supplemented with 23 µM tetracycline (Sigma-Aldrich) in a 50-ml Erlenmeyer flask at 37°C with shaking at 180 rpm. The overnight culture was then diluted into 300 ml of fresh medium to an initial OD_600_ of 0.02 and incubated under the same conditions. When cultures reached mid-log phase (OD_600_ ≈ 0.5), actinonin (50 mg/ml stock in dimethyl sulfoxide (DMSO); Sigma-Aldrich) was added to three individual 100-ml cultures at final concentrations of 0, 1, or 8 µg/ml. Cells were harvested by centrifugation (6000 × *g*, 10 min, 4°C), flash-frozen in liquid nitrogen, and stored at −80°C until further processing.

### Optimization of SILAC labeling of *E. coli* CAG12184 cells

*Escherichia coli* CAG12184 cells were cultured in RPMI (Roswell Park Memorial Institute) 1640 medium lacking Arg and Lys (Silantes GmbH, Munich, Germany) and supplemented with 2 mM alanyl-l-glutamine dipeptide (GlutaMAX, Gibco), 25 mg/l FeSO_4_·7H_2_O, 23 µM tetracycline, 1 µg/ml thiamine, 250 µM l-lysine hydrochloride, and 250 µM l-arginine hydrochloride (both light, ^12^C_6_ isotopomers). Overnight cultures were centrifuged (6000 × *g*, 5 min), the supernatant was discarded, and pellets were resuspended in fresh RPMI medium to an initial OD_600_ of 0.02. For SILAC labeling, the medium was supplemented with either 250 µM or 2 mM ^13^C_6_  l-arginine hydrochloride (Arg6) (Cambridge Isotope Laboratories, Andover, MA, USA) or 2 mM ^12^C_6_  l-arginine hydrochloride as a control. Cultures were propagated for ~2, 4, or 7 cell doublings. Cells from 50 ml cultures were harvested, and lysates were prepared as described above. Aliquots containing 200 µg of protein were transferred to protein low-binding tubes (Eppendorf) and digested overnight at 37°C with sequencing-grade modified trypsin (Promega, Madison, WI, USA) at an enzyme:substrate ratio of 1:50 (w/w) with shaking at 200 rpm. Digests were acidified to 0.1% TFA and centrifuged (16 000 × g, 10 min) to remove insoluble material. For solid-phase extraction, 100 µg of peptides in 100 µl of 0.1% TFA were processed using C18 reversed-phase tips (Bond Elut OMIX 100 µl C18, Agilent, Santa Clara, CA, USA). Tips were conditioned with 50:50 water:acetonitrile (v/v) and equilibrated with three washes of 0.1% TFA in water. Samples were aspirated through the tips 10 times to maximize binding, washed 3 times with 0.1% TFA in 98:2 water:acetonitrile (v/v), and eluted in 0.1% TFA in 30:70 water:acetonitrile (v/v). Eluates were vacuum-dried and redissolved in 100 µl of 2 mM tris(2-carboxyethyl)phosphine (TCEP) in 2% acetonitrile. Two microliters of each sample was subjected to LC-MS/MS. Light/heavy SILAC ratios were determined from MS spectra using Mascot Distiller Quantitation software (version 2.2.1), and isotope incorporation efficiency was calculated as: incorporation efficiency = [1/(ratio + 1)] × 100. After seven cell doublings in RPMI medium, median incorporation efficiencies were 96% (*n* = 156 SILAC peptide ratios from identified peptides) for cultures supplemented with 250 µM ^13^C_6_  l-arginine and 99% (*n* = 142 peptide ratios) for those supplemented with 2 mM ^13^C_6_  l-arginine.

### TRAINSPOTTER enrichment of PDF-deformylated bacterial protein N-termini following SILAC labeling

*Escherichia coli* CAG12184 cells, cultured in RPMI medium as described above, were diluted to an initial OD_600_ of 0.02 in 100 ml and grown to mid-log phase (OD_600_ ≈ 0.5). At this point, ^13^C_6_  l-arginine hydrochloride (final concentration 2 mM) and actinonin (1 µg/ml) were added and incubation was continued for 1 h (OD_600_ ≈ 0.85) or 2 h (OD_600_ ≈ 1.1). From the primary COFRADIC fractions (collected prior to *in vitro* PDF treatment), 5% aliquots were vacuum-dried and reconstituted in 100 µl of 2 mM TCEP in 2% acetonitrile. LC-MS/MS analysis was performed with 2 µl injections, and heavy/light SILAC ratios were calculated using MaxQuant (version 2.6.4.0). Isotope incorporation efficiencies were determined as described above.

### Actinonin minimum inhibitory concentration determination

MIC (minimum inhibitory concentration) values for *E. coli* WT (K-12) and the TolC-deficient CAG12184 strain (CGSC7437; F-, λ-, *tolC210*::Tn10, *rph-1*) were determined in LB and RPMI media. Cultures were inoculated at an initial OD_600_ of 0.02 in 150 µl volumes in 96-well plates (Greiner Cellstar, cat. no. 655180) and exposed to a two-fold dilution series of actinonin (17 concentrations ranging from 200 µg/ml to 0.002 µg/ml) or control medium. Plates were sealed with gas-permeable Micropore tape (3M, 1 inch wide, product no. 1530) and incubated overnight at 37°C under shaking (180 rpm) conditions. Overnight OD_600_ values were recorded in a Spark Cyto 400 multimode plate reader (Tecan, Mechelen, Belgium) controlled by SparkControl v4.0 software. Actinonin (Sigma, cat. no. A6671) was dissolved in ethanol at 20 mg/ml, with a maximum final concentration of 1% ethanol in the highest drug condition; equivalent ethanol concentrations were included in control wells.

MIC values were inferred using non-linear regression and statistical testing in GraphPad Prism 10 (v10.6.0). Specifically, one-way ANOVA with Dunnett’s multiple comparisons test was applied to compare growth at each concentration with the untreated control, with adjusted *P*-values of ≤.05 considered significant. MIC was defined as the lowest concentration at which no significant growth was detected relative to the control.

In these assays, MIC values for *E. coli* WT K-12 exceeded 200 µg/ml in both LB and RPMI conditions. In contrast, *E. coli* CAG12184 cells exhibited MICs of 0.4 µg/ml in both LB and RPMI under shaking conditions (Supplementary Fig. S3), representing >500-fold increased sensitivity compared to the WT K-12 background. These findings are consistent with a previous study reporting an MIC_90_ of 0.25 µg/ml for CAG12184 after 4 h of growth [44].

### Growth curve analysis in LB and SILAC-compatible RPMI medium

To generate growth curves of *E. coli* CAG12184, a single colony was inoculated into 3 ml of LB medium and cultured overnight at 37°C with shaking at 180 rpm. Cells were harvested by centrifugation (6 000 × *g*, 5 min, room temperature), washed twice with sterile phosphate-buffered saline, and resuspended in either LB or SILAC-compatible RPMI medium supplemented as required. Cultures were adjusted to an initial OD_600_ of 0.02 in 50 ml volumes within 250-ml Erlenmeyer flasks and incubated at 37°C with shaking at 180 rpm. For actinonin treatment, cultures were grown in their respective media until mid-log phase (OD_600_ ≈ 0.5), at which point actinonin (50 mg/ml stock in DMSO) was added to final concentrations of 1 µg/ml or 8 µg/ml. Control cultures received an equivalent volume of DMSO. Growth was monitored for ~400 min, with OD_600_ values recorded at defined intervals using a densitometer (Biowave Cell Density Meter CO 8000, WPA). Specific growth rates (*μ*) and doubling times (Td) were calculated using the Doubling Time Computing tool (Roth, 2006; http://www.doubling-time.com), which applies least-squares linear regression to the natural logarithm of OD_600_ versus time, weighting all data points equally. Calculations were restricted to the exponential growth phase (OD_600_ ≈ 0.10–0.50 before actinonin addition, and OD_600_ ≈ 0.50–1.00 post-addition). Doubling times were derived from *μ* according to the equation T_d_ = ln(2)/*μ* [45].

### COFRADIC-based enrichment of PDF-deformylated bacterial protein N-termini

Cell pellets from 50 ml cultures (stored at −80°C) were cryogenically pulverized using a liquid nitrogen-cooled mortar and pestle. The frozen material was resuspended in 1 ml of ice-cold lysis buffer (50 mM NH_4_HCO_3_, pH 7.9) and thawed on ice. Mechanical cell disruption was performed by three cycles of freeze–thawing followed by sonication (2 min total; 20-s bursts on ice at 40% duty cycle, output level 4; Branson Sonifier 250; Ultrasonic Convertor). Lysates were clarified by centrifugation (16 000 × *g*, 15 min, 4°C), and protein concentrations were determined using the DC Protein Assay Kit (Bio-Rad) according to the manufacturer’s instructions. Yields were ∼1 mg total protein per 10 ml culture at OD_600_ = 1.0. For each setup, 4 mg of protein was processed as described in Staes *et al*. [46]. Briefly, Cys residues were reduced and alkylated, and primary free amines were acetylated using the N-hydroxysuccinimide ester of stable isotopically encoded acetate (^13^C_2_D_3_-NHS-acetate) to distinguish between *in vivo* and *in vitro* acetylation (^13^C_2_D_3_-Ac or heavy Ac). Any potential O-acetylation of Ser, Thr, or Tyr residues was reverted by hydroxylamine treatment. The modified protein mixture was then digested overnight with sequencing-grade modified trypsin [Promega, Madison, WI, USA; enzyme/substrate, 1/100 (w/w)], and N-terminal peptides were enriched by SCX according to Staes *et al*. [46], with the following modifications: enzymatic liberation of pyroglutamyl peptides was omitted, and uniform methionine oxidation prior to the first RP-HPLC run was not performed. Acidified peptide mixtures (100 µl of 0.5% TFA, corresponding to 1 mg original protein input) were injected onto a Zorbax® 300SB-C18 narrow-bore column (2.1 mm ID × 150 mm, 5 µm particle size; Agilent) installed on an Agilent 1100 series capillary pump equipped with a 100 µl/min flow controller. After a 10-min isocratic run with solvent A {10 mM ammonium acetate in water:acetonitrile [98:2 (v/v)], pH 5.5}, a linear gradient was applied at 1% solvent B {10 mM ammonium acetate in acetonitrile:water [70:30 (v/v)], pH 5.5} increase per minute. The flow rate was maintained at 80 µl/min. Primary RP-HPLC fractions (4 min each) were collected between 20 and 80 min post-injection, corresponding to the peptide elution window of the gradient. Fractions were vacuum-dried and resuspended in 100 µl of 50 mM HEPES (pH 7.5) and 5 mM NiCl_2_, and incubated for 2 h at 37°C with recombinant PDF (1 µM). After acidification, fractions were re-chromatographed under identical RP-HPLC conditions. Secondary fractions were collected as follows: 1 min wide for the interval −20 to 0 min relative to the original fraction window and 30 s wide for the interval 0 to +5 min (for peptides eluting between 10 and 81 min of the gradient only). Both off-diagonal fractions (PDF-shifted, deformylated N-termini) and diagonal fractions (unshifted peptides) were collected for LC–MS/MS analysis. To reduce analytical time, secondary fractions eluting 12-min apart were pooled, yielding 90 final samples, which were vacuum-dried, resuspended in 20 µl of 2% acetonitrile, and analyzed by LC–MS/MS analysis.

### LC-MS/MS analysis and database searching

LC-MS/MS analysis of COFRADIC samples was performed using an Ultimate 3000 RSLC Nano HPLC (Dionex, Amsterdam, the Netherlands) in-line connected to an LTQ Orbitrap Velos mass spectrometer (Thermo Fisher Scientific, Bremen, Germany), as described previously [27]. Raw MS data were processed using MaxQuant (version 2.6.4.0) [47] with the Andromeda search engine [48]. Each raw dataset was searched twice, once under “specific” enzyme specificity and once under “semi-specific free N-terminus” settings. For both searches, the enzyme was defined as endoproteinase Arg-C/P (Arg-C specificity with cleavage allowed at arginine–proline sites). The “specific” searches allowed up to one missed cleavage, whereas the “semi-specific” searches allowed free N-termini and included two custom modifications to account for retention of an additional iMet or its oxidized form, thereby enabling identification of iMet-retaining N-termini as proxies for near-cognate translation initiation. Database searches were performed against both the *E. coli* (strain K-12) UniProt reference proteome (Proteome ID: UP000000625) and a six-frame translation (6-FT) database of *E. coli* generated as described in Ndah *et al*. [27]. In brief, the 6-FT database was constructed by traversing the genome in all six reading frames, identifying NTG (N = A, T, C, G) start codons and extending ORFs to the nearest in-frame stop codon (TAA, TGA, or TAG). ORFs shorter than 30 nt were discarded, redundant entries were removed, and all non-ATG starts were converted to methionine, yielding 120 714 entries. In addition, the contaminant database distributed with MaxQuant (contaminants.fasta) was included. Searches were carried out with a precursor mass tolerance of 10 ppm for the initial search (used for nonlinear mass recalibration) and 4.5 ppm for the main search. Carbamidomethylation of Cys and heavy acetylation of Lys side chains [acetyl:2H(3)C13 [2], K; not C-terminal] were set as fixed modifications. Variable modifications included Met oxidation to methionine-sulfoxide, N-terminal formylation of iMet [CO, applied to (M)], N-terminal acetylation restricted to iMet-compatible residues (Ala, Cys, Gly, Pro, Ser, Thr, Val, Met) [11, 49], heavy N-terminal acetylation {acetyl:2H(3)C13 [2], applied to any peptide N-termini [-]}, and two custom modifications enabling iMet addition (C_5_H_9_NOS) or oxidized iMet addition (C_5_H_9_NO_2_S) at free N-termini. The minimum peptide length was set at 7 aa, with a maximum of 40 for unspecific searches. The decoy strategy was “revert,” and the FDR for both peptide and protein identification was controlled at 1%. The minimum Andromeda score was set to 40 for both modified and unmodified peptides.

Results from both the UniProt and 6-FT database searches, performed under “specific” and “semi-specific free N-terminus” settings, were combined. To uniformly map the identified peptides from the different searches, mapping to the 6-FT proteome was performed using ProteoMapper (version 1.6) [50], after which only unique N-terminal peptide identifications were retained. Peptides were accepted only if they were consistent with the established rules of iMet processing [11, 49], while also considering potential initiation at non-AUG codons. Specifically, iMet processing was considered valid for N-terminal peptides beginning with iMet followed by Ala, Cys, Gly, Pro, Ser, Thr, Met, or Val, and only when the iMet was encoded by ATG or the near-cognate start codons CTG, GTG, or TTG, or in case of non-cognate upstream TIS (uTIS), those with MS evidence supporting iMet retention.

### Prediction of RNA secondary structure

A 48-nt window spanning −30 to +15 nt relative to, and including, the start codon was extracted for each TIS from the TRAINSPOTTER *E. coli* dataset [i.e. 688 database-annotated TISs (dbTISs) and 118 aTISs]. As a null distribution independent of TIS contexts, 668 control RNA sequences of length 48 nt were generated using a first-order (dinucleotide) Markov model estimated genome-wide from the *E. coli* K-12 MG1655 reference genome (RefSeq assembly GCF_000005845.2); sequences were simulated with a fixed random seed and converted to the RNA code. Minimum free energies (MFEs) were predicted with RNAfold (ViennaRNA Package v2.7.0) under default settings (Turner energy parameters, 37°C) and MFEs (kcal/mol) were parsed from the program output. Distributions were visualized as Tukey box-and-whisker plots (box = 25th–75th percentiles; median line; whiskers = 1.5 × IQR) with individual values overlaid using horizontal jitter. Group differences among dbTISs, aTISs, and random sequences were assessed by a two-sided Kruskal–Wallis test followed by Dunn’s multiple-comparisons procedure with adjusted *P*-values reported. Graphs were produced in GraphPad Prism (v10.6.0).

### Motif frequency analysis at TIS contexts

For each identified TIS, a 60-nt sequence window (−30 to + 27 nt relative to the start codon) was extracted from the *E. coli* genome. Within these sequences, occurrences of AGG and GGA (SD-like motifs) and AAA (A-rich motifs) were counted in a position-specific manner, considering overlapping triplets. The relative frequency of each motif at every position was expressed as the percentage of sequences containing the respective triplet starting at that position. Frequencies were then averaged across all sequences within each group—TIS harboring an SD-like motif (AGG, GGA) within an effective ribosome-binding-site (RBS) spacing window (3–15 nt upstream of the start codon) and TISs lacking such motifs. As a baseline control, randomly sampled internal ATG codons from annotated coding sequences (excluding the first 30 nt of each CDS) were extracted in a strand-aware manner and analyzed using the same 60-nt window and motif-counting procedure. The resulting positional frequencies were plotted as heatmaps (Fig. 5), showing the distribution of SD-like and A-rich motifs around the TIS.

### Homology modeling of RibE N-terminal proteoforms (SWISS-MODEL)

The amino acid sequences of *E. coli* RibE^L^, RibE^M^, and RibE^S^ were submitted to SWISS-MODEL (release 2025.1). The top-scoring template identified by HHblits search was PDB ID 3MK3.1.A, corresponding to the *Salmonella enterica* serovar Typhimurium lumazine synthase.

Model generation used the homo-60-mer icosahedral assembly defined by the template. Sequence alignment indicated ~90%–91% identity between the *E. coli* isoforms and the *Salmonella* template. The models achieved GMQE = 0.93 and QMEANDisCo global = 0.87 ± 0.05, indicating reliable backbone geometry and conserved quaternary arrangement. Because modeling was based on the icosahedral template, these assemblies represent structurally compatible 60-mer configurations rather than experimental evidence that the *E. coli* proteoforms assemble into 60-mers.

### Structural visualization of RibE proteoform assemblies

The SWISS-MODEL homology assemblies were analyzed and visualized in UCSF ChimeraX v1.10.1. Models were rendered as space-filling (surface) representations, with subunits colored by chain identity to highlight quaternary organization. For RibE^L^, residues 1–4 were highlighted in lime; for RibE^M^, residues 1–2; and for RibE^S^, the iMet (corresponding to Met_4_ in RibE^L^) was colored in lime to indicate the truncated N-terminus.

### AlphaFold3 multimeric modeling of RibE proteoforms

To assess the stability of the oligomeric interfaces independently of the template, each RibE proteoform sequence was submitted to AlphaFold v3 (multimeric mode) using the default multiple-sequence-alignment and template parameters. AlphaFold multimeric (AF3) predicted homopentameric assemblies with high confidence scores (*pTM* = 0.92–0.94; *ipTM* = 0.91–0.93). Per-residue confidence metrics (pLDDT) and predicted aligned error (PAE) maps were inspected to evaluate interface reliability and N-terminal flexibility. All three models exhibited well-defined β-barrel cores, whereas the N-terminal region showed lower pLDDT and elevated PAE values, indicating greater local uncertainty and potential conformational variability. For presentation, ribbon renderings were colored by pLDDT (per-residue predicted local distance difference test; blue to yellow gradient) to visualize local confidence.

### Software packages and data visualization

Data visualization was performed using GraphPad Prism (version 10.4.1; GraphPad Software) and RAWGraphs 2.0 (https://app.rawgraphs.io/).

## Results

### Introducing TRAINSPOTTER: a novel COFRADIC methodology for enriching nascent N-termini indicative of translation initiation sites

In bacteria, protein synthesis universally initiates with fMet, making Nt-formylated peptides the most direct proteomic proxies of TISs. However, because the formyl group is rapidly removed co-translationally by PDF during early translation [10], such peptides are rare at steady state [6, 51]. We developed TRAINSPOTTER—a modification of the N-terminal COFRADIC workflow—to selectively enrich formylated nascent N-termini by exploiting the predictable hydrophilic shift caused by *in vitro* PDF-mediated deformylation. This shift allows chromatographic separation of formylated from pre-existing, non-formylated N-termini, enabling confident TIS mapping.

#### Proof-of-concept: PDF-induced hydrophilic shift of synthetic peptides

Before applying the method to complex bacterial proteomes, we tested its feasibility using synthetic peptides representing distinct N-terminally modified variants. Peptides bearing an fMet (For-M), an Nt-free Met (NH_2_-M, α-amino-unmodified), an Nt-acetylated non-Met (Ace-X; a rare post-translational N-terminal modification in bacteria [52]), and an iMet-processed, Nt-free N-terminus (NH_2_-X, iMet removed) were incubated with recombinant PDF. RP-HPLC and LC-MS analyses following PDF treatment confirmed that only the Nt-formylated peptides underwent quantitative deformylation, whereas Nt-acetylated and Nt-free peptides remained unchanged (Fig. 1B and Supplementary Figs S1 and S2A). Removal of the hydrophobic Nt-formyl group and concomitant exposure of a protonated Nt-amine produced a pronounced hydrophilic shift in retention time. Under acidic conditions (0.1% TFA, pH 3.0), deformylated peptides eluted ∼10 min earlier than their formylated counterparts (Fig. 1B), compared with ∼3 min under milder conditions (10 mM ammonium acetate, pH 5.5) (data not shown). The larger shift at lower pH reflects greater amine protonation, increasing peptide polarity and reducing hydrophobic interactions with the stationary phase.

PDF also effectively deformylated oxidized fMet (fMet sulfoxide), indicating that spontaneous methionine oxidation—while itself increasing peptide hydrophilicity—does not impede deformylation in the *in vitro* condition used (Supplementary Fig. S2B). The broad substrate specificity of PDF is a notable advantage, as *in vivo*, PDF deformylates the vast majority of nascent polypeptides regardless of the second residue (P2′), with only diminished activity reported toward acidic residues at P2′ (Asp, Glu) and exquisite selectivity for Nt-formyl over Nt-acetyl groups [53]. This promiscuity ensures unbiased targeting of diverse Nt-formyl peptides *in vitro*, maximizing coverage of nascent N-termini across the proteome. Together, these experiments establish that PDF-mediated deformylation can chromatographically separate fMet-initiated N-termini based on altered hydrophobicity, providing a quantitative and specific basis for enriching these otherwise elusive nascent N-termini.

#### TRAINSPOTTER applied to the *E. coli* N-terminal proteome

We next applied TRAINSPOTTER to complex *E. coli* TolC-deficient CAG12184 (*tolC*::Tn10) proteomes, leveraging a COFRADIC N-terminomics strategy to enrich for protein N-termini prior to the deformylation step [36]. Inactivation of the multidrug efflux channel TolC in this strain reduced the MIC of actinonin (Supplementary Fig. S3), thereby facilitating effective PDF inhibition. While TRAINSPOTTER can, in theory, detect naturally retained fMet-initiated N-termini, their extremely low abundance under normal growth conditions [6, 51] prompted us to enhance their representation through *in vivo* PDF inhibition. Within this framework, TRAINSPOTTER relies on a deformylation-dependent chromatographic shift that selectively captures previously Nt-formylated peptides, while actinonin treatment increases the detectable pool of these substrates and thereby enhances sensitivity.

**Figure 2.**
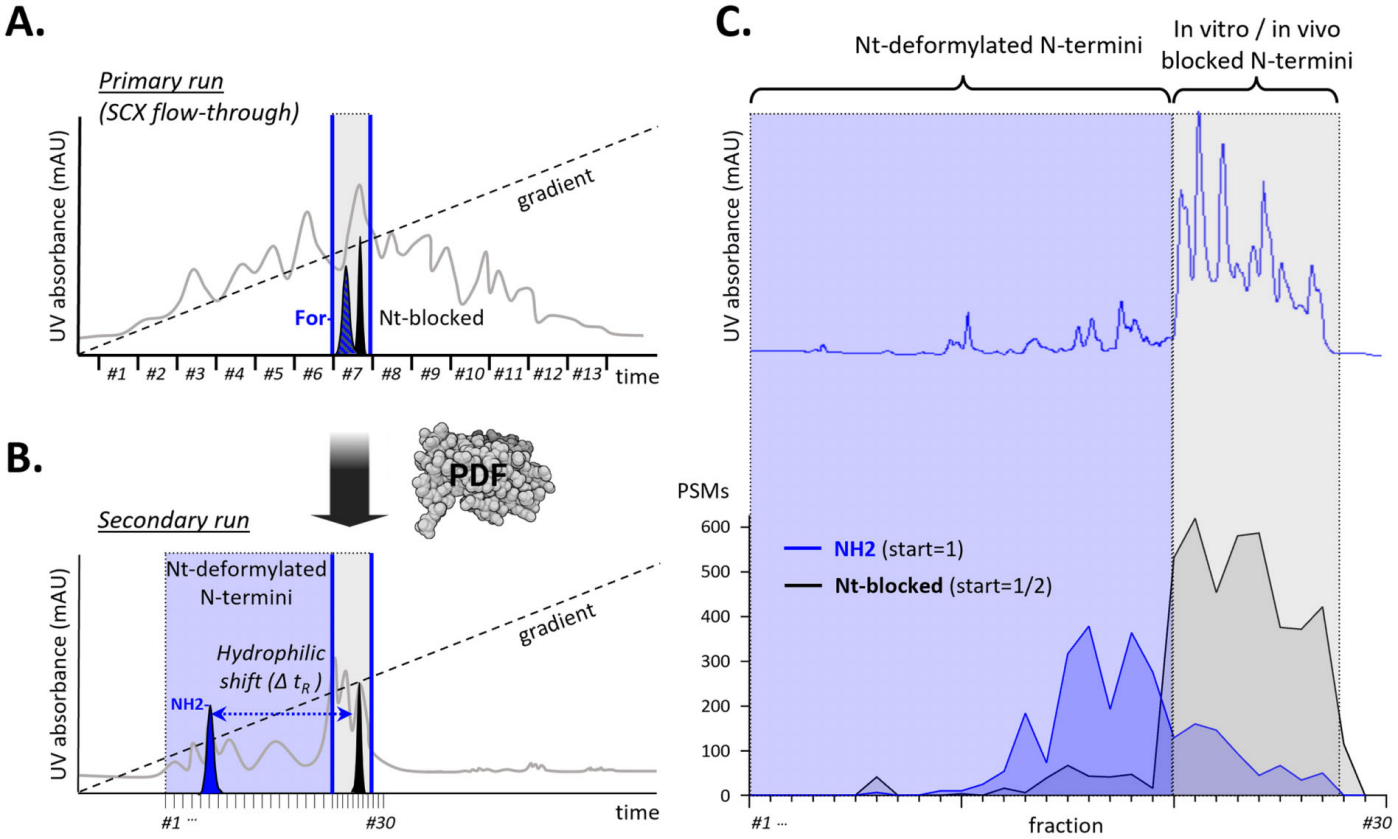
Primary and secondary COFRADIC chromatographic separations used in TRAINSPOTTER. (**A**) Schematic primary RP-HPLC UV chromatogram (214 nm; mAU) of the SCX flow-through peptide fraction enriched in N-terminal peptides. The chromatographic run is collected in consecutive 4-min fractions (fraction #); the primary fraction used for the schematic shown in panel (B) is indicated (fraction #7). (**B**) Schematic of the secondary RP-HPLC separation of the selected primary fraction following incubation with recombinant PDF. PDF-mediated deformylation of Nt-formylated peptides increases hydrophilicity, resulting in earlier elution (off-diagonal; blue shading; hydrophilic retention-time shift, Δt_R_) relative to the original primary collection interval (diagonal; gray shading), which predominantly contains *in vivo* or *in vitro* Nt-blocked N-termini. Peptides eluting up to ∼20 min before and ∼1 min after the original primary interval are collected. (**C**) Representative secondary RP-HPLC UV chromatogram (top) and corresponding LC–MS/MS identification yield per collected fraction (bottom) for a primary fraction spanning 40–44 min. Shading corresponds to the schematic regions defined in (**B**), with the early-eluting off-diagonal region (blue shading) enriched for Nt-deformylated N-termini and the diagonal region (gray shading) enriched for N-terminally blocked peptides. The lower plot shows peptide-spectrum match (PSM) counts per secondary fraction for NH_2_-Met starting N-termini mapping to start position 1 (NH_2_; start = 1; blue; *n* = 2619 PSMs) and N-terminally blocked peptides mapping to start positions 1 (*n* = 2548 PSMs) or 2 (*n* = 1832 PSMs) (Nt-blocked; start = 1/2; black).

Cells grown in LB medium were exposed for 1 h during exponential growth to a sub-lethal dose of actinonin, a potent PDF inhibitor. The working concentration reduced the growth rate to ∼70% of the untreated control (Supplementary Fig. S4B) and corresponded to 2.5× the experimentally determined bacteriostatic MIC for *E. coli* CAG12184 (Supplementary Fig. S3B), consistent with previous reports [6]. Under these transient exposure conditions, actinonin treatment “traps” fMet on nascent chains without complete growth arrest, therby increasing the yield of formylated N-termini for analysis. Briefly, following actinonin treatment, cells were harvested, lysed, and proteins processed by the first steps of a standard N-terminal COFRADIC protocol [46]: all primary amines were blocked via N-acetylation [using an isotopically labeled (^13^C_2_D_3_) NHS-acetate to distinguish *in vitro* modifications (^13^C_2_D_3_-Ac or heavy Ac)], cysteines were reduced/alkylated, and modified proteins were digested with trypsin (which, under these blocking conditions, cleaves only after Arg). Internal peptides were removed by SCX chromatography, yielding a peptide mixture highly enriched in N-terminal peptides [36, 46] (Fig. 1C). These were fractionated by first-dimension RP-HPLC, collecting 4-min fractions across the gradient (Fig. 2A).

Next, the core TRAINSPOTTER step was introduced: each primary RP-HPLC fraction was treated with recombinant PDF to reveal peptides retaining an Nt-formyl group *in vivo*. Formylated peptides eluted earlier upon re-chromatography (off-diagonal fractions in the two-dimensional chromatographic space [54]), whereas deformylated or non-formylated peptides retained their retention time (diagonal fractions) [54] (Fig. 2B and C).

An example separation is shown in Fig. 2C: a primary fraction (40–44 min) was treated with PDF, and formerly Nt-formylated peptides shifted to earlier elution in the secondary run. Both shifted and unshifted fractions were collected for LC–MS/MS analysis. The proteomics data were searched against the UniProt database and a 6-FT database of the *E. coli* genome, the latter allowing identification of N-termini indicative of non-annotated translation initiation events [27]. LC–MS/MS of shifted fractions revealed strong enrichment for Nt-free N-termini starting at protein position 1 (retaining iMet), consistent with authentic TIS-indicative peptides. In contrast, diagonal fractions were dominated by *in vivo* Nt-blocked N-termini or proteolytically processed (*in vitro* Nt-blocked) N-termini [position 2 (removal of iMet) or beyond starts] (Fig. 2C). These results demonstrate that TRAINSPOTTER, particularly when combined with *in vivo* PDF inhibition, enables selective isolation of nascent N-termini indicative of translation initiation events from complex bacterial proteomes.

### SILAC labeling confirms nascent origin of deformylated N-termini

Because the Nt-formyl modification—our direct proteomic proxy for a TIS—is intentionally removed *in vitro* during TRAINSPOTTER to generate the diagnostic chromatographic shift, the shift itself is already indicative of *in vivo* formylation. However, to provide complementary confirmation of nascent origin, we incorporated pulse SILAC (pSILAC) labeling into TRAINSPOTTER to enable quantitative discrimination between newly synthesized and pre-existing proteins.

#### SILAC labeling optimization in *E. coli*

To validate that the sorted N-termini in the TRAINSPOTTER workflow represent bona fide nascent protein N-termini (i.e. a positive enrichment of deformylated bacterial protein N-termini), we first optimized SILAC conditions for *E. coli*. SILAC has historically been applied to only a limited number of bacteria (e.g. *E. coli* [55] and *Salmonella* [56, 57]) because heavy amino acid incorporation can be complicated by metabolic conversions, particularly in prototrophic strains. Early implementations often relied on auxotrophic mutants or deletions in lysine/arginine biosynthetic pathways. However, more recent work demonstrated that supplying high concentrations of amino acids can suppress metabolic conversions and enable full heavy-label incorporation even in prototrophs [58]. Consistent with this, we found that supplementing SILAC-compatible, defined RPMI medium with 2 mM ^13^C_6_  l-arginine (Arg6) (plus essential cofactors such as lysine, vitamins, and Fe^2+^) supported robust growth of the TolC-deficient strain (Supplementary Fig. S4C) while yielding efficient uptake and incorporation of heavy Arg. After ∼7 doublings under these conditions, incorporation into Arg-containing peptides approached 99% (versus ∼96% at 0.25 mM Arg6) (Supplementary Fig. S5). No evidence of alternative heavy-label incorporation via amino acid conversion was observed, confirming the suitability of these conditions for labeling ArgC/P-type N-termini in the N-terminomics workflow.

#### Pulse SILAC application in the TRAINSPOTTER workflow

While in conventional SILAC [59] experiments for quantitative proteomics cells are cultured for ≥5 doublings in “heavy” medium to achieve complete substitution of the target amino acid, pSILAC instead introduces a pulse of (a) heavy amino acid(s) to selectively label nascent proteins, leaving pre-existing proteins light-labeled. This approach has been used to determine protein degradation rates and half-lives on a proteome-wide scale [60], and we previously used it to assess Nt-proteoform stability [38].

For implementing pSILAC in this study, the *E. coli* TolC-deficient CAG12184 strain was grown in Arg-deficient, SILAC-compatible RPMI medium to mid-log phase (OD_600_ ≈ 0.5), then pulsed with 2 mM Arg6 together with the PDF inhibitor actinonin (1 µg/ml) for 1–2 h before harvesting (Supplementary Fig. S4C). This pulse labels proteins synthesized during the incubation with heavy Arg, enabling direct discrimination of newly synthesized peptides from the pre-existing (light-labeled) proteome.

#### Distinct SILAC incorporation profiles for formylated versus processed peptides

**Figure 3.**
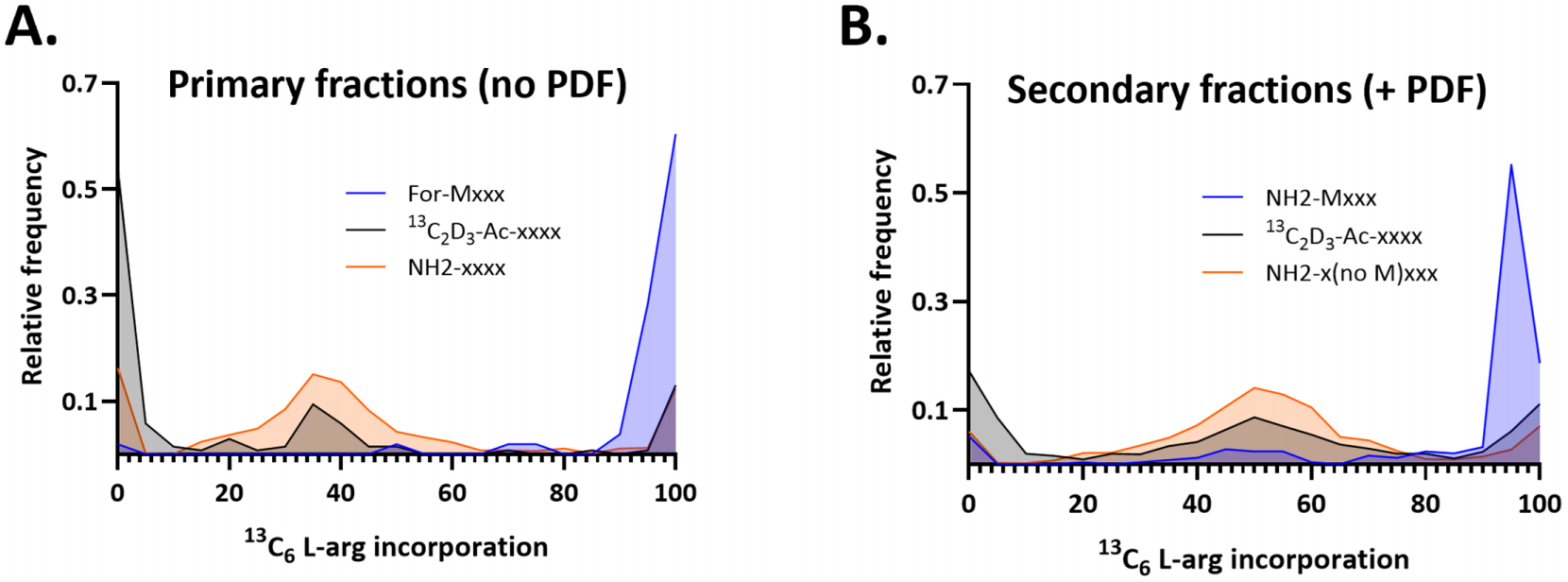
SILAC incorporation profiles for distinct peptide classes during COFRADIC-based enrichment of PDF-deformylated bacterial protein N-termini in *E. coli* TolC-deficient CAG12184. SILAC incorporation efficiencies were determined for three peptide classes identified in (**A**) primary COFRADIC fractions collected before *in vitro* peptide deformylation by the action of PDF and (**B**) secondary COFRADIC fractions collected after PDF treatment. *E. coli* CAG12184 cells were grown in RPMI medium to an OD_600_ of 0.5, after which ^13^C_6_  l-arginine hydrochloride (Arg6) (final concentration 2 mM) and actinonin (1 µg/ml) were added. Cultures for panel (A) were incubated for an additional 1 h (final OD_600_ ≈ 0.85) prior to harvesting, while cultures for panel (B) were incubated for an additional 2 h (OD_600_ ≈ 1.1) prior to harvesting. Light/heavy SILAC ratios for all identified peptides were calculated using MaxQuant, and incorporation efficiency was determined as: incorporation efficiency = [1/(ratio + 1)] × 100. For each peptide class, the distribution of mean incorporation per unique peptide was plotted by binning values in 5% increments. Incorporation profiles display relative frequency (*Y*-axis) versus SILAC incorporation (*X*-axis) as line plots, with shaded areas indicating the distribution for each class. Panel (A): Nt-formylated peptides (For-Mxxx; blue, *n* = 53; median = 100%, mean = 94%), Nt-heavy-acetylated peptides (^13^C_2_D_3_-Ac-xxxx; black, *n* = 138; median = 1.5%, mean = 24%), and Nt-free, α-amino-unmodified (Nt-free) peptides (NH_2_-xxxx; orange, *n* = 850; median = 37%, mean = 41%). Panel (B): Nt-free, α-amino-unmodified peptides starting with methionine (NH_2_-Mxxx; blue in line with their Nt-formylated precursors, *n* = 252; median = 95%, and mean = 84%), Nt-heavy-acetylated (^13^C_2_D_3_-Ac-) black (black, *n* = 657; median = 49%, mean = 47%), and Nt-free, α-amino-unmodified peptides not starting with methionine (orange, *n* = 1587; median = 52%, mean = 53%). In panel (B), Nt-free peptides starting with methionine—corresponding to shifted, deformylated peptides—exhibit low light/heavy ratios (indicative of high heavy-label incorporation), consistent with their nascent origin and matching the labeling pattern of Nt-formylated peptides in panel (A). These findings confirm that the TRAINSPOTTER workflow specifically enriches newly synthesized bacterial N-termini in the shifted secondary COFRADIC fractions.

By coupling pSILAC to the TRAINSPOTTER workflow, we quantitatively compared heavy-label incorporation across distinct N-terminal peptide classes. Figure 3 shows the ^13^C_6_-Arg incorporation distributions for peptides identified in the primary COFRADIC fraction (before *in vitro* deformylation; Fig. 3A) and in the secondary fraction (after *in vitro* PDF treatment; Fig. 3B).

In the primary fraction (1 h labeling), N-terminally formylated peptides (initiator fMet present *in vivo*) had a median 100% heavy incorporation (mean 94%), indicating that virtually all originated from proteins synthesized during the pulse. In contrast, N-termini that were unmodified *in vivo* and thus chemically N-acetylated during the workflow (Nt-heavy-acetylated) showed minimal heavy incorporation after 1 h of labeling (median ∼1.5%, mean ∼24%), reflecting an “older” pre-pulse proteoform pool. Free, α-amino-unmodified peptides (Nt-free) showed intermediate incorporation (median ∼37%, 1 h labeling), consistent with a mixture of old and new protein forms (roughly one-third newly synthesized during the pulse) (Fig. 3A).

In the secondary fraction (2 h labeling), heavy-label enrichment was most pronounced for shifted-deformylated N-termini—those originally Nt-formylated *in vivo* and converted to free N-termini only upon *in vitro* PDF treatment. Nt-free peptides starting with methionine (predominantly shifted-deformylated N-termini with the iMet retained) were overwhelmingly heavy-labeled (median ∼95%, mean ∼84%). Other secondary-fraction peptides, such as Nt-free peptides not starting with Met (median ∼52%) or Nt-heavy-acetylated peptides (median ∼49%), showed lower incorporation, consistent with a roughly even mixture of new and old proteins after ∼2 h of growth (Fig. 3B). The clear separation in incorporation profiles demonstrates that the TRAINSPOTTER workflow selectively enriches nascent, deformylation-dependent N-termini in the shifted fraction.

**Figure 4.**
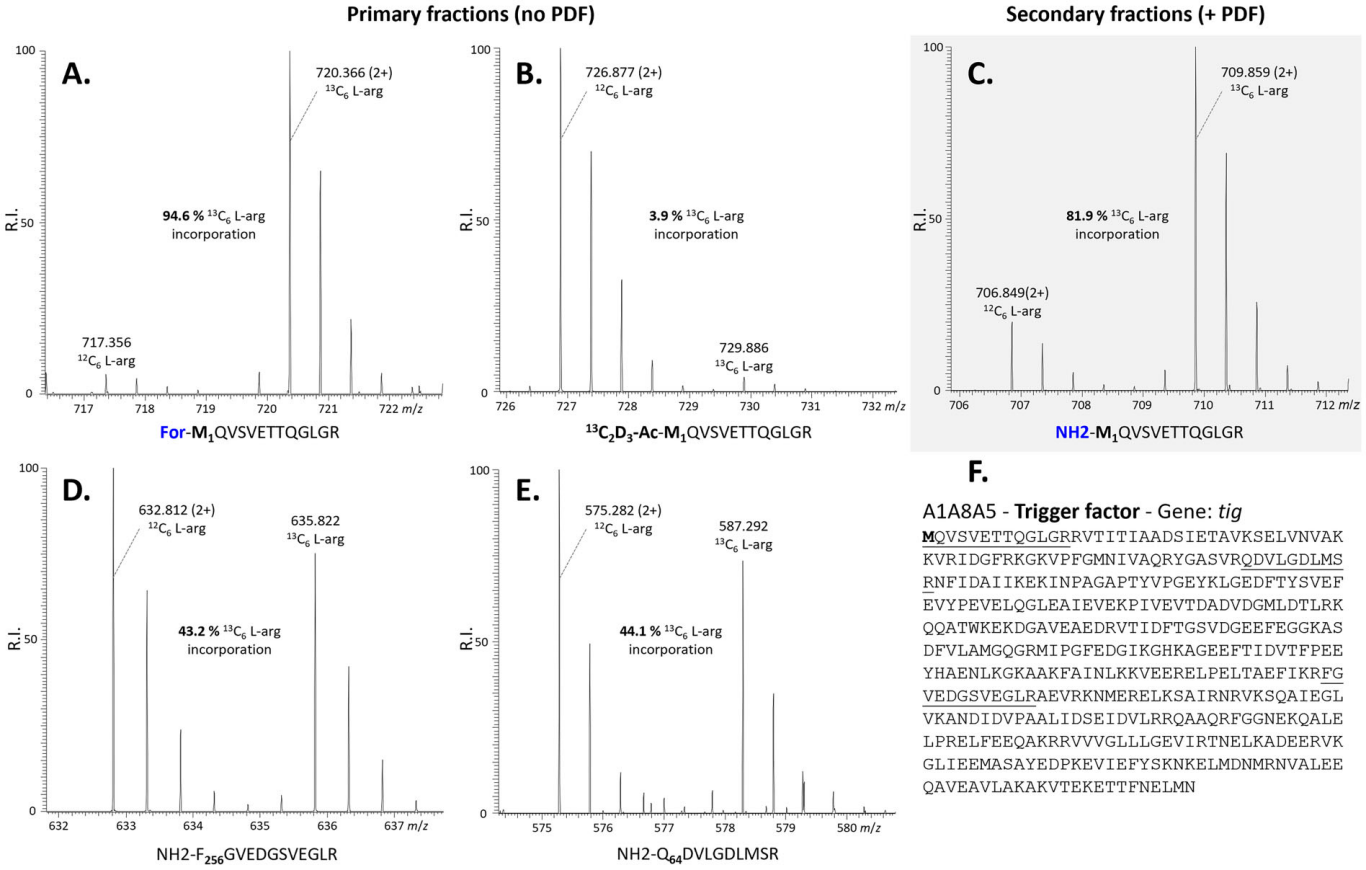
Representative MS spectra of *E. coli* Trigger factor (Tig) peptides from distinct (N-terminal) peptide classes. Representative MS spectra are shown for peptides belonging to distinct (N-terminal) peptide classes. All peptides originate from the *E. coli* protein Trigger factor (UniProt accession A1A8A5, gene name *tig*). Panel (**A**) shows the N-terminally formylated N-terminal peptide (For-M_1_QVSVETTQGLGR) identified in a primary COFRADIC fraction collected before *in vitro* peptide deformylation by the action of PDF, exhibiting 94.6% ^13^C_6_  l-arginine incorporation (1 h labeling)—consistent with the mean incorporation for Nt-formylated peptides in Fig. 3A (median = 100%, mean = 94%). Panel (**B**) shows the heavy Nt-acetylated (^13^C_2_D_3_-Ac-) counterpart of the N-terminal peptide (^13^C_2_D_3_-Ac-M_1_QVSVETTQGLGR), also from a primary COFRADIC fraction, with 3.9% incorporation, matching the low labeling levels observed for Nt-heavy-acetylated peptides in Fig. 3A (median = 1.5%, mean = 24%). Panel (**C**) displays the Nt-free, α-amino-unmodified N-terminal peptide starting with methionine (M_1_QVSVETTQGLGR) identified in secondary COFRADIC fractions after PDF treatment. This shifted-deformylated peptide shows a low light/heavy ratio (2 h labeling), indicative of high heavy-label incorporation, consistent with its nascent origin and closely matching the labeling pattern of its Nt-formylated precursor in panel (A). Panels (**D**) and (**E**) show two internal tryptic ArgC/P peptides (F_256_GVEDGSVEGLR and Q_64_DVLGDLMSR), with incorporation efficiencies of 43.2% and 44.1%, respectively—values in line with Nt-free, α-amino-unmodified peptides in Fig. 3A (median = 37%, mean = 41%). Panel (**F**) maps all identified peptides (underlined) from panels (A)–(E) onto the Trigger factor protein sequence. Cultures corresponding to panels (A), (B), (D), and (E) were grown in RPMI medium to OD_600_ ≈ 0.5, supplemented with 2 mM ^13^C_6_  l-arginine hydrochloride and 1 µg/ml actinonin, and incubated for an additional 1 h (final OD_600_ ≈ 0.85) prior to harvesting, whereas the culture for panel (C) was harvested after 2 h (final OD_600_ ≈ 1.1). Together, these data highlight the substantially higher contribution of recent translation to the formylated N-terminal peptide compared with both internal peptides and indicate that the Nt-heavy-acetylated N-terminal peptide predominantly represents a pool derived from earlier translation events, with only a minor fraction reflecting recent synthesis. This also confirms that the shifted-deformylated peptide in panel (C) retains the nascent ^13^C_6_  l-arginine (Arg6) labeling pattern characteristic of its formylated precursor.

Representative MS spectra illustrate these quantitative patterns. For instance, in Trigger factor (Tig) (Fig. 4), the formylated N-terminus (For-M_1_QVSVETTQGLGR) was almost fully heavy-labeled (∼94.6%) (Fig. 4A), whereas the chemically heavy-acetylated *in vivo* deformylated counterpart (^13^C_2_D_3_-Ac-M_1_QVSVETTQGLGR) showed only ∼3.9% incorporation (Fig. 4B). The shifted-deformylated peptide (M_1_QVSVETTQGLGR) retained high incorporation (∼81.9%) (Fig. 4C), matching the nascent profile of its formylated precursor (Fig. 4A). Similar trends were observed for 30S ribosomal protein S9 RpsI (Supplementary Fig. S6), where the *in vitro* PDF-generated nascent N-terminus showed >94% heavy incorporation (Supplementary Fig. S6A), the *in vivo* deformylated form had intermediate labeling (∼44.8%) (Supplementary Fig. S6B), and the iMet-processed proteoform exhibited minimal labeling (∼2%) (Supplementary Fig. S6C). Internal peptides for both proteins showed ∼38%–44% heavy incorporation (Supplementary Fig. S6D), representing the level of steady-state proteome labeling (Fig. 3). The stepwise decrease in heavy-label incorporation from the nascent fMet terminus to the deformylated species and, ultimately, to the iMet-processed proteoform mirrors the canonical processing order: initiation with fMet, PDF-mediated deformylation, and subsequent MetAP-dependent iMet removal.

These examples, consistent across proteins examined, confirm that deformylated N-termini captured via *in vitro* PDF treatment in the shifted COFRADIC fraction originate from actively translated, nascent chains. Consequently, Nt-free, α-amino-unmodified Met-starting N-terminal peptides identified in the shifted fractions serve as robust, peptide-level proxies for genuine bacterial translation initiation events.

### The (alternative) translation initiation site landscape

To enable direct comparison with previously reported bacterial TIS landscapes derived from Ribo-seq analyses [25, 27, 61, 62], we examined in detail the TRAINSPOTTER dataset generated from *E. coli* cells grown in LB medium and treated during the exponential phase for 1 h with sublethal concentrations of actinonin. For this analysis, we considered only N-termini that complied with established iMet-processing rules [11, 49] and originated from (near-)cognate start codons or, in the case of non-cognate upstream TIS (uTIS), those with MS evidence supported iMet retention supporting their authenticity as genuine Nt-proteoforms [19].

**Figure 5.**
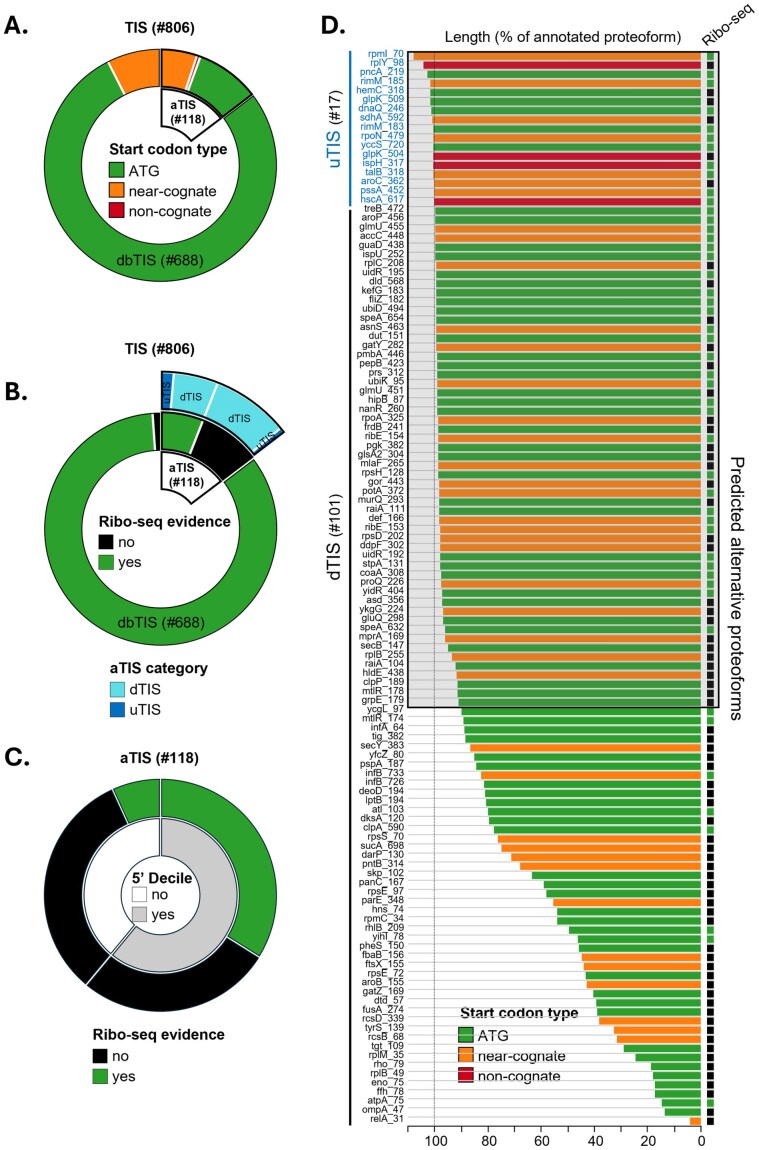
Integrated overview of (alternative) translation initiation sites identified by TRAINSPOTTER in *E. coli*. (**A**) Donut chart showing the distribution of start codon types for all identified TISs (*n* = 806), subdivided into dbTISs (*n* = 688) and aTISs (*n* = 118). Start codon classes are color-coded as ATG (green), near-cognate (orange), and non-cognate (red), highlighting the increased contribution of non-ATG start codons among aTISs. (**B**) Donut chart summarizing Ribo-seq support for dbTISs and aTISs. aTISs are further subdivided into upstream (uTIS) and downstream (dTIS) categories, illustrating that alternative initiation sites—particularly dTISs—are less frequently supported by Ribo-seq compared with annotated dbTISs (Supplementary Table S1). (**C**) Donut chart showing Ribo-seq support for aTISs (*n* = 118) stratified by genomic position relative to the annotated start codon. aTISs located within the first decile (5′ proximal downstream or upstream region) of the coding sequence exhibits higher Ribo-seq support than more distal dTISs, indicating a positional bias in Ribo-seq detectability and/or initiation efficiency. (**D**) Horizontal bar chart plot summarizing 108 unique aTISs, ordered by the relative length of the aTIS-initiated proteoform compared to the UniProt-annotated database proteoform (expressed as % of annotated length). Each horizontal bar represents a unique aTIS; corresponding gene names are followed by the inferred N-terminal proteoform length (aa). Bar color indicates start codon identity (ATG, green; near-cognate, orange; non-cognate, red). Light gray shading highlights aTISs located within the first decile (5′ proximal region) of the annotated coding sequence. uTISs (*n* = 17) are highlighted in blue. Ribo-seq annotations (squares to the right of each bar) indicate whether the corresponding aTIS is supported by Ribo-seq evidence (green, supported; black, no support).

In total, 1082 unique N-termini indicative of translation initiation were identified, mapping to 806 distinct TISs across 729 proteins (Supplementary Table S1 and Fig. 5). These findings reveal both the prevalence of alternative TIS (aTIS) usage—defined as experimentally supported TISs that do not correspond to dbTISs—and the frequent co-expression of multiple Nt-proteoforms per gene.

Of the 1082 unique N-termini, 953 (88%) mapped to amino acid position 1 or 2, corresponding to 688 UniProt dbTIS. Among these dbTISs, 628 (91%) originated from ATG start codons and 60 (9%) from near-cognate codons (50 GTG, 10 TTG). The remaining 129 N-termini initiated from start codons located either downstream (dTIS) or upstream (uTIS) and in-frame with the annotated start, corresponding to 118 aTISs (118 of 806 unique TIS coordinates, 14.6%) and reflecting the presence of alternative Nt-proteoforms. Among these, 17 uTISs (17 N-termini) produced N-terminally extended proteoforms, represented by six ATG codons (35.3%), seven near-cognate codons (six ATG and one ATC; 41.2%), and four non-cognate codons (23.5%) with proteomic evidence of iMet recoding. In addition, 112 N-termini corresponded to downstream TISs (dTISs), mapping to 101 sites represented by 67 ATG codons (66.3%) and 34 near-cognate codons (33.7%). Over half of these dTISs (52 sites) generated truncated proteoforms differing by fewer than 15 amino acids (aa) compared to the annotated full-length counterpart, including 37 cases with ≤5 aa differences. This observation is consistent with previous reports showing that alternative initiation frequently occurs at closely spaced start codons [19, 25] (Supplementary Table S1 and Fig. 5).

Comparison with existing Ribo-seq datasets [25, 27, 61, 62] revealed strong concordance. Of the 806 TISs identified by TRAINSPOTTER, 658 (82%; 913 N-termini) precisely matched the coordinates as defined by retapamulin-assisted ribosome profiling (Ribo-RET). An additional 59 TISs (73 N-termini) coincided with initiation sites inferred from conventional Ribo-seq elongation data but not detected by Ribo-RET. In total, 717 TISs (986 N-termini; 89%) were supported by Ribo-seq, with a further 10 TISs (10 N-termini) showing near Ribo-RET coordinate matches (±3 codons) (Supplementary Table S1 and Fig. 5).

Overall, only 79 TISs (10%; 86 N-termini) identified by TRAINSPOTTER lacked Ribo-seq support. Of these, 9 dbTISs (12 N-termini; 11%) were unsupported, whereas the majority (74 N-termini; 70 aTISs; 89%) represented non-database-annotated alternative initiation events. Notably, although 45 non-database N-termini (38 aTISs) were supported by Ribo-seq, the largest unsupported category still comprised aTISs (Fig. 5).

Conversely, when considering dbTISs detected in a ribosome profiling dataset generated under comparable conditions [25], distinct ribosome-density peaks were observed at annotated start codons for 991 of 1153 expressed genes (∼86%), whereas TRAINSPOTTER recovered 688 dbTISs (∼60% of expressed genes). In line with previous riboproteogenomics efforts, this difference in sensitivity can largely be attributed to known proteomics-related biases, including reduced sensitivity for low-abundance proteins, loss of initiating N-termini due to signal-peptide cleavage, and cases in which N-terminal peptides are inherently not amenable to MS detection [19, 20, 27, 33, 63, 64].

### Local mRNA folding stability around annotated and alternative TISs

**Figure 6.**
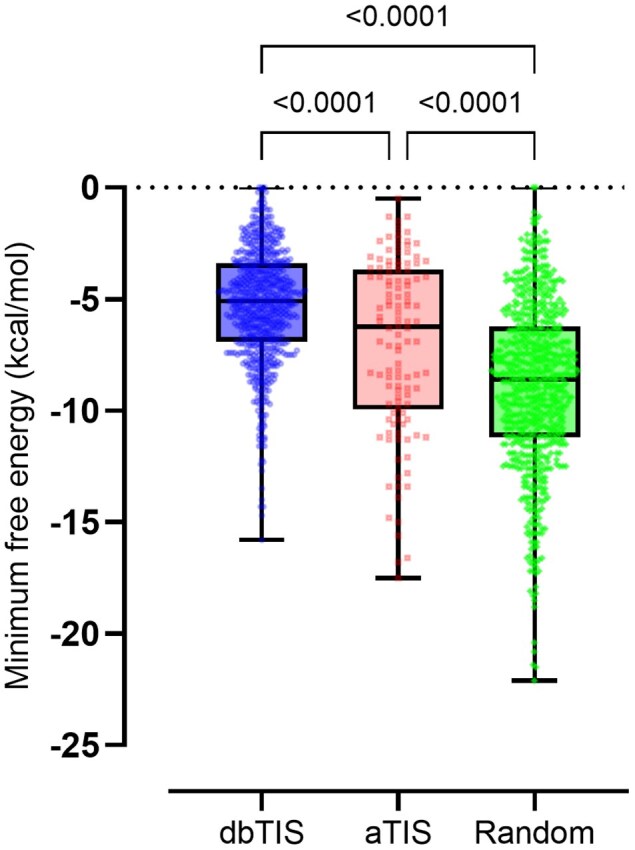
Predicted RNA folding stability around annotated and alternative *E. coli* TISs. Box-and-whisker plots of MFE for 48-nt windows (−30 to +15 nt relative to the start codon; start codon included) centered on dbTISs (*n* = 688), aTISs (*n* = 118), and genome-based random controls of the same length (*n* = 668). Boxes show the interquartile range (IQR; 25th–75th percentile) with the median as a horizontal line; whiskers are Tukey (1.5 × IQR). Individual MFEs are plotted as jittered points. dbTIS windows have a slightly higher MFE than aTIS windows (medians: −5.1 versus −6.25 kcal/mol), whereas random sequences are substantially more stable (median ≈ −8.6 kcal/mol). Both dbTISs and aTISs are significantly less stable than random, and aTISs are modestly more stable than dbTISs (two-sided Kruskal–Wallis with Dunn’s multiple-comparisons; adjusted *P*-values shown above brackets; all comparisons *P* < .0001 in this panel). Medians (IQR): dbTIS −5.1 (−6.9 to −3.4), aTIS −6.25 (−9.85 to −3.7), and random ≈ −8.6 (−11.2 to −6.275) kcal/mol.

Since stable mRNA secondary structures can influence translation initiation (efficiency), we next analyzed predicted folding energies across 48-nt windows (−30 to +15 nt) centered on annotated and alternative start codons. aTISs exhibited slightly more negative MFEs than annotated TISs, indicating modestly stronger local structure. Nevertheless, both classes were significantly less structured than randomized genomic controls (median MFEs: −5.1 kcal/mol for dbTISs, −6.25 kcal/mol for aTISs, and −8.6 kcal/mol for random sequences) (Fig. 6). This relative depletion of secondary structure near both annotated and alternative start sites is consistent with these regions being initiation-competent.

### Ribosome-binding site features of Shine–Dalgarno-dependent and -independent TISs

**Figure 7.**
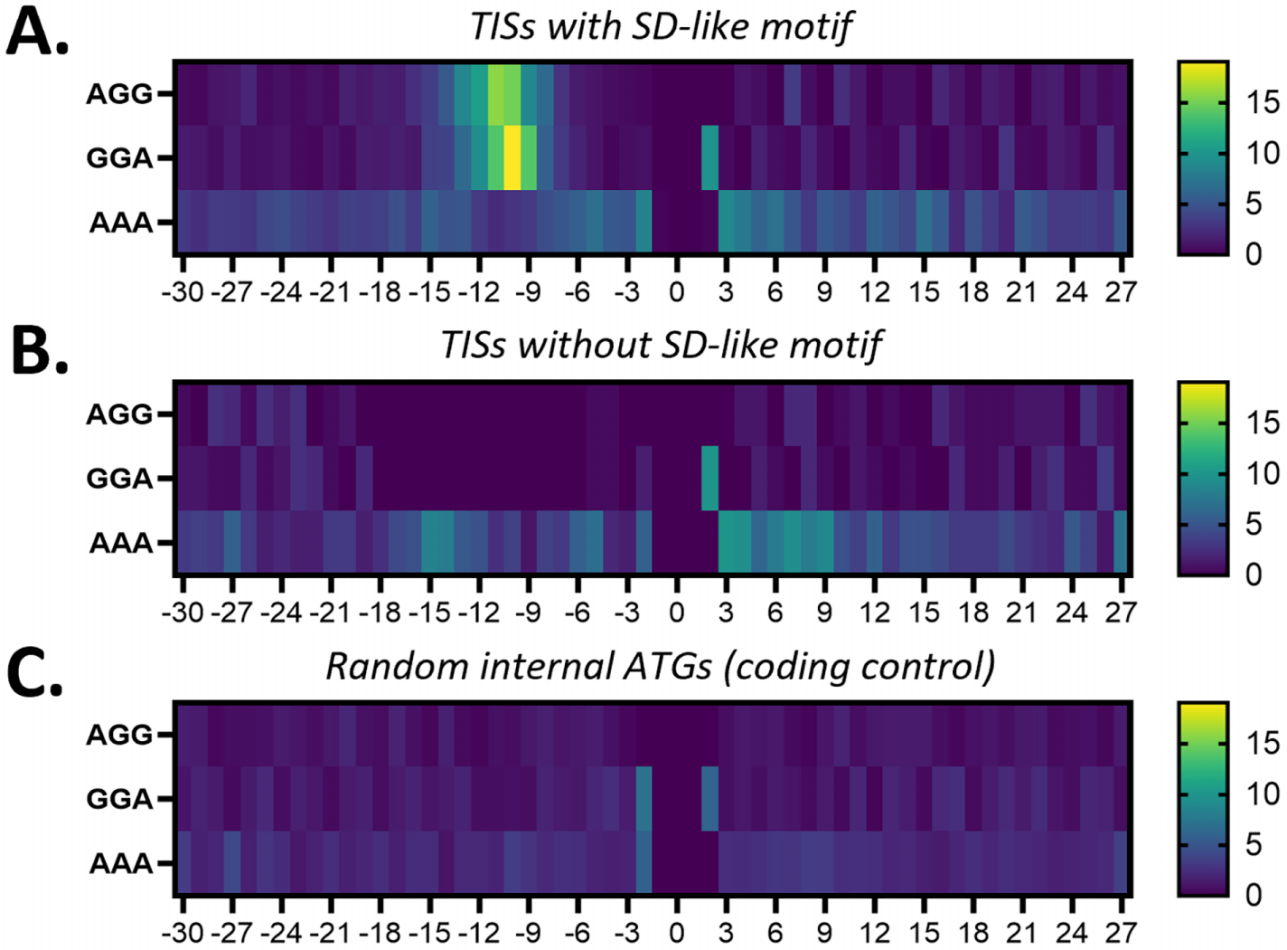
Positional enrichment of SD-like and A-rich trinucleotide motifs around identified TISs and random ATGs in *E. coli*. Heatmaps show the positional frequency (% occurrence per position) of AGG and GGA (SD proxy motifs) and AAA (A-rich motif) relative to the first nucleotide of the identified start codon (position 0, corresponding to the first nt of the TIS). Each position corresponds to the start of the trinucleotide within a 60-nt window (−30 to +27 nt) centered on the TIS. (**A**) SD-containing TISs. Heatmap for 614 unique TISs containing at least one SD-like motif (AGG or GGA) within an effective RBS spacing window, defined here as 3–15 nt upstream of the start codon. A spacing of ∼5–10 nt generally represents the optimal distance between the 3′ end of the SD sequence and the start codon, facilitating base pairing with the 3′ terminus of the 16S rRNA and proper positioning of the start codon in the ribosomal P-site [65–67]. (**B**) SD-lacking TISs. Heatmap for 192 unique TISs lacking any SD-like motif within the same upstream spacing window. The distribution of the AAA motif highlights A-rich regions previously reported to enhance translation initiation efficiency [68], typically occurring within 17–3 nt upstream and +1 to +6 nt downstream of the start codon. (**C**) Random internal ATG control sites. Heatmap for randomly sampled internal ATG codons from annotated coding sequences, excluding the first 30 nt of each CDS to avoid true initiation regions (*n* = 614; random seed = 42). For each control ATG, an identical 60-nt window (−30 to +27 nt) was extracted in a strand-aware manner. Color scale indicates the frequency of each trinucleotide at each position (%, as shown).

Analysis of sequence context around identified TISs revealed distinct initiation signatures (Fig. 7). Among the 806 TISs defined by TRAINSPOTTER, 614 contained at least one Shine–Dalgarno (SD)-like motif (AGG or GGA) within an effective RBS spacing window of 3–15 nt upstream of the start codon, corresponding to the region enabling base pairing between the SD sequence and the 3′ terminus of the 16S rRNA for accurate start codon positioning in the ribosomal P-site [65–67]. The majority of these were dbTISs (564), while 50 represented aTISs. In contrast, 192 TISs (124 dbTISs and 68 aTISs) lacked upstream SD-like motifs (Supplementary Table S1). Nevertheless, both SD-dependent and SD-independent TISs displayed enrichment of A-rich (AAA) motifs within the canonical regions typically associated with translation initiation [68] (Fig. 7). Specifically, both SD-containing and SD-lacking TISs exhibited clear A-rich enrichment, consistent with previous reports showing that AAA triplets are preferentially enriched between −17 and −3 nt upstream and +1 to +6 nt downstream of the start codon [68]. Importantly, comparison with randomly sampled internal ATG codons from coding regions (Fig. 7C) showed that this A-rich enrichment is not observed in random ATG contexts. Mean AAA frequencies were comparable between the two groups, indicating that adenosine-rich sequence context is a shared feature of translation initiation regions. Thus, even in the absence of canonical SD sequences, the upstream and downstream compositions of these regions conforms to established characteristics of initiation-competent sites, supporting their classification as genuine translation initiation events.

### Co-expressed N-terminal proteoforms are common and biologically suggestive

Since several of the alternative Nt-proteoforms identified in this study lacked complementary N-terminal peptide evidence for expression of their annotated counterpart or were unsupported by Ribo-seq data, caution is warranted when interpreting genes proposed to encode multiple Nt-proteoforms. For example, the 7-aa-truncated PotA proteoform detected here (Supplementary Table S1) matches a previously identified Ribo-seq TIS, where the absence of a Ribo-RET signal at the annotated site was interpreted as evidence of potential misannotation [25]. However, similar assumptions proved unreliable—for example, the annotated *pmbA* TIS, also deemed likely false based on lacking Ribo-RET signal, yet clear N-terminal evidence (despite low ribo-RET signal) for this annotated start, was independently confirmed in *Salmonella* [19].

To ensure robust interpretation, we therefore focused on genes with complementary N-terminal peptide evidence supporting two or three in-frame initiation sites, representing the most confident cases of co-expressed Nt-proteoforms.

Across the TRAINSPOTTER dataset (Supplementary Table S1), 68 genes showed distinct N-terminal peptide evidence supporting two or three in-frame initiation sites, corresponding to the expression of multiple Nt-proteoforms from a single gene (i.e. proteoform pairs/triplets) (Supplementary Table S2). This group included several well-characterized examples, such as *infB*, encoding translation initiation factor IF2. *infB* uses three in-frame start codons (primary AUG and internal GUG_158_ and AUG_165_) to produce IF2 isoforms with different N-termini [69]. Although all IF2 proteoforms bind the ribosome and promote fMet-tRNA recruitment, they are not functionally redundant: both major isoforms, IF2-1 (also referred to as IF2α or IF2^L^, initiating at AUG_1_) and IF2-2 (IF2β or IF2^M^, initiating at GUG_158_), are required for optimal growth, with single-isoform strains exhibiting reduced growth rates and cold sensitivity. Notably, the longer IF2-1 isoform possesses an N-terminal extension absent in IF2-2; this segment has been implicated in stress-related processes such as DNA repair, as only IF2-1 facilitates replication restart following DNA damage [70, 71]. The third start codon, AUG_165_, gives rise to IF2-3 (IF2β′ or IF2^S^) [69]. Together, the three IF2 isoforms differentially modulate DNA recombination efficiency, influencing the rate of synapsis between homologous DNA molecules [72].

Another well-characterized example is *speA*, which encodes biosynthetic arginine decarboxylase. In addition to the canonical full-length form, we detected the previously reported N-terminally truncated SpeA proteoform (SpeA^S^), lacking 26 aa [19, 25], which has been implicated in differential subcellular compartmentalization [25]. Moreover, an additional proteoform, SpeA^M^, initiating four residues downstream of this variant was identified, further expanding the set of plausible, differentially localized SpeA proteoforms present *in vivo*.

Other reported cases of proteoform pair-encoding genes identified here include *clpA*, in which ClpA^S^ (supported by identification of an N-terminal peptide matching an aTIS at AUG_169_) represses Clp self-proteolysis by acting as a decoy [73, 74], and the *hemC*-encoded proteoform pair [19].

**Figure 8.**
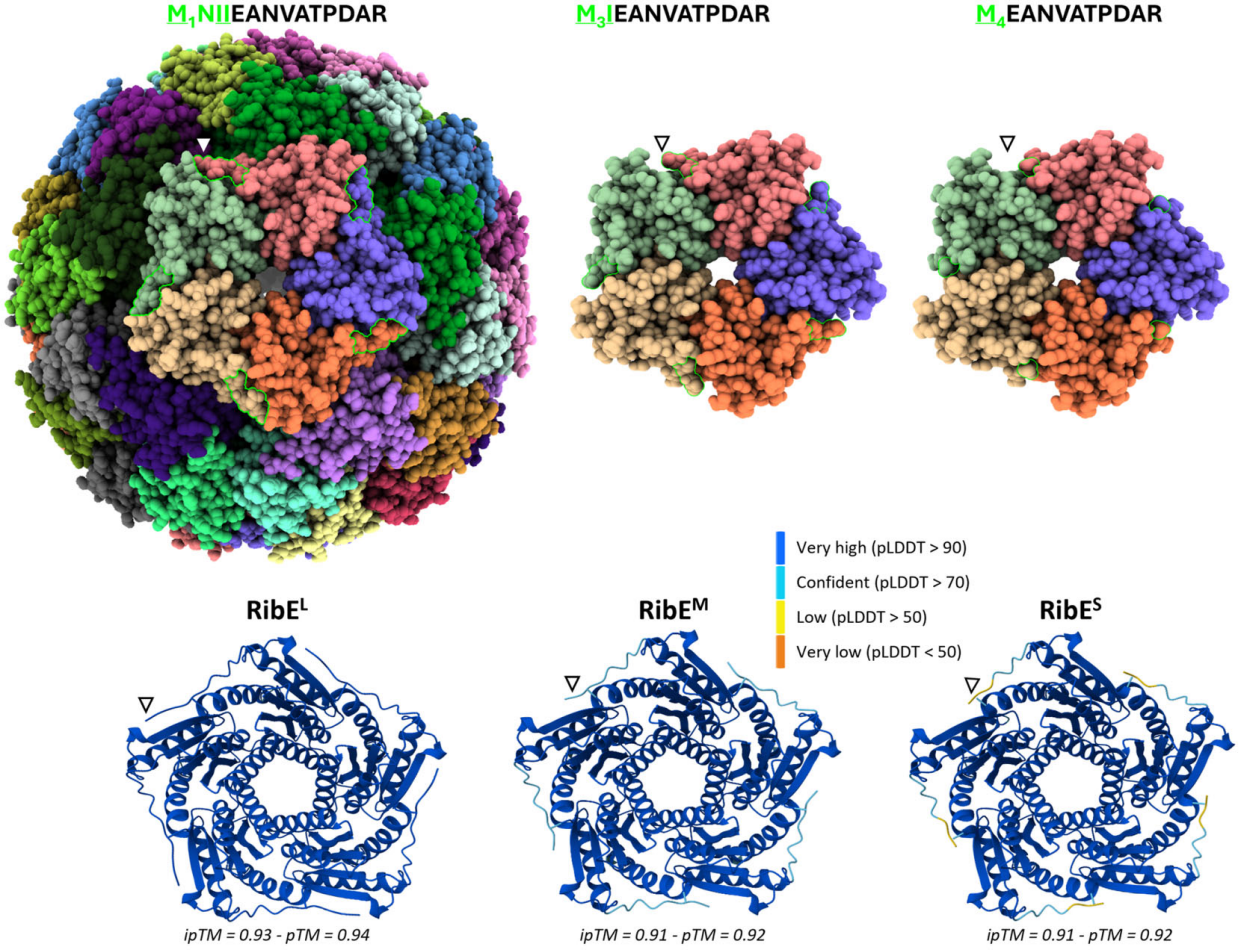
RibE N-terminal proteoforms validated by TRAINSPOTTER and their predicted structural consequences. Identified RibE N-terminal peptides defining each of the three identified translation initiation start sites are shown: MNIIEANVATPDAR for the long (L) proteoform (RibE^L^, detected with heavy N-terminal acetylation), MIEANVATPDAR for the middle (M) proteoform (RibE^M^, iMet retained), and MEANVATPDAR for the short (S) proteoform (RibE^S^, iMet retained). Upper panels: homology models of *E. coli* RibE proteoforms were generated in SWISS-MODEL using the *S. enterica* serovar Typhimurium lumazine synthase template 3MK3.1.A (sequence identity 90%–91%). Because SWISS-MODEL builds on the 60-subunit icosahedral template, each *E. coli* sequence could be modeled within that quaternary framework, yielding plausible homo-60-meric icosahedral assemblies, characteristic of bacterial lumazine synthases. However, this modeling reflects the structural compatibility of the monomers with the icosahedral scaffold rather than experimental evidence that these proteoforms assemble into 60-mers. Model quality metrics (GMQE = 0.93; QMEANDisCo ≈ 0.87 ± 0.05) indicate high confidence in the monomeric fold and the modeled inter-subunit geometry. The left panel shows the predicted RibE^L^ 60-mer, with residues 1–4 highlighted in lime to mark the full-length, intact N-terminus. The middle and right panels display one representative pentameric block from the modeled truncated RibE^M^ and RibE^S^ proteoforms, respectively, initiating from downstream start codons and lacking two or three N-terminal residues. The corresponding first residues of each truncated proteoform are shown in lime. All subunits are colored identically to emphasize structural similarity among proteoforms. Bottom panels: AlphaFold3 (multimeric mode) oligomer predictions for RibE^L^, RibE^M^, and RibE^S^ yield high-confidence homopentameric assemblies (pTM 0.92–0.94; ipTM 0.91–0.93). Relative to RibE^L^, the truncated forms show reduced inter-chain confidence and elevated PAE near the N-terminal contact region, together with lower per-residue confidence scores (pLDDT) and decreased secondary-structure definition in this zone. These differences suggest that N-terminal shortening weakens pentamer–pentamer interactions and may destabilize the icosahedral 60-mer. Ribbon representations are colored by pLDDT (higher values indicate greater local confidence). AF3, AlphaFold v3; pTM, predicted TM-score; ipTM, inter-chain predicted TM-score; PAE, predicted aligned error.

Beyond these previously described cases, the dataset revealed several additional interesting genes with multiple in-frame initiation sites that may have functional consequences. For instance, *ribE*, encoding lumazine synthase, was found to produce multiple proteoforms initiated at an AUG and two near-cognate ATT codons. Lumazine synthase assembles into a 60-subunit icosahedral capsid, and while its flexible conserved N-terminal segment (∼10 residues) does not contribute directly to the catalytic β-barrel core, it plays a key role in inter-subunit contacts and quaternary structure formation [75, 76]. Consequently, truncation of this N-terminal arm is therefore expected to destabilize the *E. coli* enzyme, leading to disassembly of the 60-mer capsid into pentamers or altered higher-order assembly. AF3 models of the three identified N-terminal RibE proteoforms support this notion that, while each forms a stable pentamer (ipTM ≈ 0.91–0.93), progressive N-terminal shortening reduces interface confidence in the inter-chain contact zone (Fig. 8). This reduction in local confidence implies increased flexibility or partial disorder of the N-terminal arm, which in lumazine synthases contributes to subunit packing and icosahedral stability. Accordingly, the shorter N-termini are predicted to yield weaker inter-subunit interactions and a greater tendency toward partial disassembly into pentameric intermediates. These results suggest that alternative initiation may fine-tune RibE complex stability.

Similarly, for *raiA (yfiA)*, four N-terminal peptides—MTMNITSKQMEITPAIR, TMNITSKQMEITPAIR, MNITSKQMEITPAIR, and MEITPAIR—resolved to three distinct TIS (the annotated start codon plus initiations at codons 3 and 10). The first two peptides correspond to iMet-retaining and iMet-processed forms from the dbTIS. The N-terminal domain of RaiA forms the principal ribosome-binding interface, and residues upstream of Met10 contribute to intramolecular stabilization and contacts with 16S rRNA (PDB: 4Y4O) [77, 78]. Truncation of this region is therefore expected to alter the N-terminal fold and reduce ribosome affinity. Consistent with this, AlphaFold models indicate that the shorter variants remodel the N-terminal arm (data not shown), potentially weakening RaiA-ribosome interactions and decreasing stabilization of inactive 70S ribosomes. Since, within the bacterial ribosome hibernation pathway, RaiA favors monomeric 70S particles, whereas the ribosome modulation factor (RMF) promotes 70S dimerization and the hibernation-promoting factor (HPF) stabilizes the resulting 100S complexes, the truncations identified here may shift the equilibrium toward RMF/HPF-driven 100S ribosome formation [78].

Beyond these examples, other multi-proteoform genes also suggest potential N-terminal functional tuning. In addition to *infB, speA, ribE*, and *raiA*, triplet Nt-proteoform evidence was found for *glmU, glpK*, and several ribosome-related genes, including *rimM, rpsE*, and *rplB*. Overall, genes encoding ribosomal proteins were enriched among those showing distinct N-terminal peptide evidence for multiple in-frame initiation sites.

Among these, two ribosomal protein genes—*rplY* (L25) and *rpmI* (L35)—illustrate how alternative initiation may fine-tune translation under autoregulatoryq control. *rplY* harbors a cryptic initiation codon (AGA) located 12 nt upstream of the annotated AUG, giving rise to an N-terminally extended L25 proteoform, supported by the identification of the iMet-retaining N-terminal peptide (MEKEM_1_FTINAEVR). Similarly, *rpmI* can initiate at a GTG codon five codons upstream of its canonical start, producing an L35 variant elongated by five residues (MEVIKM_1_PKIKTVR). Both genes are subject to tight translational feedback control: L25 binds to a structured element within the 5′ UTR of its own mRNA to repress initiation [79], whereas L20 binds a long-range pseudoknot that overlaps the *rpmI* start codon to inhibit L35 synthesis [80]. The upstream initiation sites identified here likely function as low-efficiency “leak” points that permit limited translation when primary initiation is repressed—either by L25- or L20-mediated feedback or during the nutrient deprivation-induced stringent response—thereby maintaining basal synthesis of these essential ribosomal proteins. In the case of *rpmI*, the upstream GTG codon occurs just before the structured operator that mediates L20-dependent repression. Initiation at this site may locally unwind or bypass this inhibitory RNA structure, allowing basal L35 translation under repressive conditions. Consistent with this interpretation, the *rplY* leader includes an atypical SD-like motif and a conserved secondary structure despite permitting high basal translation efficiency [79].

In summary, *rplY* and *rpmI* likely employ upstream translation initiation as a translational tuning strategy, mitigating the impact of feedback controls and contributing to homeostatic regulation of ribosome assembly components.

Altogether, this set of 68 genes encoding multiple Nt-proteoforms underscores how alternative initiation can diversify the bacterial proteome at single-gene resolution, suggesting that N-terminal variability—even by only a few residues—can influence a protein’s localization, interaction network, or assembly state.

## Discussion

The TRAINSPOTTER methodology represents a complementary proteomic advance to Ribo-seq in mapping bacterial translation initiation, directly enabling the identification of nascent protein N-termini through a distinctive biochemical signature. By strategically leveraging the universal N-terminal formylation of iMet in bacteria, optionally combined with *in vivo* PDF inhibition and subsequent unbiased *in vitro* deformylation, this approach provides direct proteomic evidence for translation initiation events. In contrast to Ribo-seq approaches that infer initiation from ribosome footprints, limiting precise delineation of initiation codons, particularly when multiple potential start sites are closely spaced [19, 25–27], TRAINSPOTTER reads out peptide-level markers of start-site identity and resolves closely spaced initiation sites with single-amino-acid precision, thereby helping to clarify ambiguous genome annotations and Nt-proteoform complexity.

Compared with a related COFRADIC-based proteogenomic workflow combining N-terminal enrichment and PDF inhibition to chart the translation initiation landscape of *Listeria monocytogenes* [41], TRAINSPOTTER permits the analysis of both shifted and non-shifted peptide fractions. This dual analysis enables the characterization of complementary N-terminal species—formylated, deformylated, and iMet-processed N-termini—thereby providing richer and more comprehensive evidence for Nt-proteoform diversity. In contrast to general MS-based N-terminomics workflows that enrich protein N-termini irrespective of their origin (e.g. translational versus proteolytic [34, 81]), TRAINSPOTTER is specifically designed to resolve nascent, initiation-derived N-termini by exploiting the transient formylation state unique to bacterial translation initiation. This selectivity arises from the deformylation-dependent chromatographic shift, which enables the specific capture of previously Nt-formylated peptides and directly links peptide-level detection to translation initiation.

To further contextualize TRAINSPOTTER performance relative to prior Nt-formylation-focused N-terminomics studies, at the level of Nt-formylation-supported dbTISs, substantial concordance is observed between datasets, with ∼80% of Nt-formylation-supported sites reported by Bienvenut *et al*. [6] also detected here, while TRAINSPOTTER increases overall coverage by approximately two-fold, identifying Nt-formylation-supported evidence for >500 annotated dbTISs (Supplementary Table S1). This increased coverage is consistent with the selective enrichment of nascent, initiation-derived N-terminal peptides achieved through deformylation-assisted chromatographic separation, a selectivity that is further supported by complementary lines of evidence, including the selective *in vitro* deformylation of Nt-formylated synthetic peptides and pSILAC-based confirmation of nascent protein synthesis. Consistent with this, the set of TISs identified by TRAINSPOTTER closely matches those reported by complementary Ribo-seq strategies [25, 27, 61, 62]. Antibiotic-stalled Ribo-seq variants such as Ribo-RET and tetracycline-inhibited profiling (TetRP) have enabled genome-wide maps of initiating ribosomes [25, 26], revealing that alternative start usage is pervasive in bacteria. For example, a landmark Ribo-RET study in *E. coli* uncovered dozens of genes showing in-frame alternative initiation, providing a first global glimpse of “alternative proteomes” encoded within conventional genes [25]. Proteomic data validate and extend these observations: most alternative N-termini detected correspond to initiation events evident—if not fully resolved—in prior Ribo-RET, TetRP, or related datasets. This concordance indicates that TRAINSPOTTER captures bona fide initiation events rather than artifacts. Moreover, by delivering peptide-level confirmation of start-site identity—including cases initiated from near-cognate (e.g. GTG, TTG) and even non-cognate codons—TRAINSPOTTER provides definitive evidence for translation initiation events that would be challenging to predict or distinguish using sequence-based or ribosome-footprint analysis alone. The ability to directly “read out” these biochemical signatures underscores the power of deformylation-assisted N-terminomics for elucidating the true bacterial N-terminome.

Notably, the agreement between our N-terminal evidence and Ribo-seq calls is substantially higher than in prior reports. This improvement reflects (i) aggregating initiation evidence across multiple Ribo-seq datasets [25, 27, 61, 62] rather than relying on a single study and (ii) harmonizing calling rules that often differ between pipelines (e.g. how closely spaced initiation peaks are merged or filtered, the accepted start-codon set, and minimum coverage/abundance thresholds), underscoring how algorithmic choices shape Ribo-seq-inferred TIS sets.

Importantly, *in vitro* Ribo-seq or INRI-seq (cell-free Ribo-RET) validated ∼70% of annotated *E. coli* TISs [61] and contributed substantially to the increased overlap with our proteomic TIS landscape. A key caveat for discovering aTISs, however, is the current design of INRI-seq, which captures only the start codon plus the first 50 codons. Consequently, aTISs beyond this initial segment cannot be called. In practice, our broader synthesis of Ribo-RET evidence—each dataset with distinct sensitivity and design constraints—reduces apparent discordance driven by study-specific thresholds rather than biology, explaining the higher overlap we observe and highlighting the need for improved, standardized TIS-calling strategies.

TRAINSPOTTER yields nascent-specific molecular evidence: heavy-isotope pulse labeling confirmed that deformylation-shifted peptides were predominantly nascent, with high heavy-label incorporation clearly distinguishing them from the pre-existing N-terminome. In practice, any N-terminal peptide detected with an unmodified iMet and high heavy incorporation almost certainly derives from an authentic start site of a nascent protein. When the same protein also carried an *in vivo*-processed N-terminus (e.g. iMet removal or Nt-acetylation), we typically observed the mature N-terminal peptide as a separate species with much lower heavy labeling, reflecting a pre-existing proteoform. This one-to-one correspondence between nascent and mature proteoforms (e.g. formylated versus deformylated/iMet-processed versions of Tig or RpsI (Fig. 4 and Supplementary Fig. S6) demonstrates that the shifted N-termini arise from active translation rather than proteolysis, and that TRAINSPOTTER can resolve alternative initiation products alongside co-translational processing *in vivo*, adding a critical layer of confidence to proteogenomic discovery of alternative proteoforms. We note, however, that this interpretation depends on active synthesis during the pulse window. For example, the N-terminal peptide M_1_AVQQNKPTR from the large ribosomal subunit protein bL32 (RpmF) was observed in its formylated form, exclusively light-labeled in the primary fraction, and its iMet-processed counterpart (AVQQNKPTR) likewise appeared only in the light state (Supplementary Table S1). Together, these observations indicate that under the sampled conditions, RpmF was not appreciably synthesized during the pulse; the signals derive from the pre-existing proteoform pool rather than nascent translation.

We included actinonin-mediated PDF inhibition to enrich formylated nascent peptides, acknowledging that such perturbation affects physiology [15, 82]. PDF inhibition serves to increase the detectable pool of Nt-formylated substrates and thus enhances sensitivity, whereas its omission primarily reduces detection depth without affecting the underlying separation principle. Despite this perturbation, the translation initiation landscape closely mirrored that of untreated *E. coli*, and its strong concordance with Ribo-RET supports the physiological relevance of the detected initiation events. Importantly, since formylated N-termini are intrinsically present in bacterial proteomes, albeit often at low abundance [6, 41], the workflow can also be run without PDF inhibition—e.g. to minimize perturbation in sensitive settings or to profile under defined physiological or stress conditions—while still allowing nascent N-terminome profiling. Reduced sensitivity under such conditions may be mitigated by sampling multiple growth conditions, including faster growth regimes where increased translational flux is expected to elevate the abundance of nascent protein N-termini, increasing analytical depth through more sensitive MS acquisition strategies, or applying alternative enrichment workflows targeting blocked N-termini. As an intermediate strategy, pulsed or transient PDF inhibition may further enhance detection of initiation-derived N-termini while limiting cellular perturbation.

The deformylation-assisted workflow also opens avenues for exploring biological phenomena that remain largely inaccessible to current proteomic methods. Certain proteins, notably membrane-associated or very small proteins, may (partially) evade co-translational deformylation due to restricted accessibility of PDF. For membrane proteins, the nascent chain may become embedded or targeted before PDF can access the N-terminus, while microproteins may complete synthesis entirely before deformylation occurs, leading to (partial) N-terminal formyl retention [83, 84]. Indeed, a small fraction of bacterial proteins has been reported to remain formylated at steady state [41, 44]. TRAINSPOTTER provides a direct means to systematically identify and characterize such formylated proteoforms and, when applied under controlled conditions, to chart how formyl retention varies across proteins and environments. Moreover, since microproteins are historically under-represented owing to their small size [20], their higher likelihood of formyl retention may render them particularly amenable to detection with this strategy. Consistent with this, we identified the N-terminus of the 17-aa *mgtA* leader peptide MgtL, as well as 86 TISs corresponding to 79 sORFs encoding microproteins (<100 aa). These represent ~11% of all genes detected here—comparable to their overall genomic prevalence (12.6%) (with no enrichment expected under conditions of general PDF inhibition)—highlighting that TRAINSPOTTER effectively captures microprotein initiation events that are typically underrepresented in proteomic datasets.

A notable limitation of N-terminomics, including TRAINSPOTTER, is its inability to capture TIS-indicative N-termini of proteins that undergo signal peptide cleavage. Unlike, for example, iMet removal—which can be readily detected—signal peptide cleavage occurs during Sec or Tat pathway-mediated membrane translocation, rendering the preprotein N-terminus inaccessible to detection due to its fast turnover [85]. Since over 10% of UniProt-annotated *E. coli* proteins are predicted to contain cleavable signal peptides, their initiation sites typically escape detection by N-terminal proteomics. In contrast, Ribo-RET readily captures these TISs. We identified only five such annotated N-termini—TcyJ and OppA (predicted Sec/SPI substrates), DmsA and HybO (Tat/SPI), and YdhY (TATLIPO)—despite high SignalP-6.0 cleavage probabilities (>99% for all but TcyJ at 83%) (Supplementary Table S1), suggesting rare detection of preprotein precursors or, less likely, mispredicted signal peptides.

In addition, TRAINSPOTTER may underrepresent initiation events that produce unstable protein products, as detection requires that the nascent N-terminal peptide persists long enough to be captured by the workflow. While Ribo-seq is inherently blind to post-translational protein stability, N-terminal proteomics provides a complementary perspective that selectively reports on initiation events yielding peptides with at least transient stability, as supported by our pulse-SILAC validation.

More generally, quantitative labeling strategies such as SILAC also provide a conceptual framework to assess proteoform stability and turnover, as previously demonstrated using positional proteomics in human cultured cells, where N-terminal proteoform stability could be inferred from labeling dynamics and turnover showed a modest positive correlation with protein abundance [38]. While such analyses were not the focus of the present study, they highlight the potential of combining N-terminal proteomics with quantitative labeling to explore initiation-site-specific proteoform stability. Importantly, proteoform stability is highly responsive to cellular context—particularly in bacteria—suggesting that sampling across multiple growth conditions may further increase the likelihood of capturing conditionally unstable translation products [86].

It is also instructive to contrast TRAINSPOTTER with metabolic labeling approaches such as BONCAT and O-propargyl-puromycin tagging [87–89]. While those methods enrich newly synthesized proteins, they do not inherently pinpoint start-site identity. In contrast, TRAINSPOTTER focuses on the iMet peptide itself—the minimal defining unit of a proteoform—offering both start-site specificity and compatibility with quantitative, high-resolution MS.

Beyond its methodological contributions, TRAINSPOTTER has broad implications for genome annotation and bacterial proteome complexity. The long-held “one gene, one protein” paradigm is increasingly obsolete; many bacterial genes encode multiple protein products via alternative translation initiation [29, 90]. N-terminal proteomic evidence provides direct confirmation of this principle, reinforcing evidence that extensive Nt-proteoform diversity exists across bacterial species. Importantly, these alternative proteoforms can display distinct subcellular localizations or biological functions, effectively partitioning a single gene’s output into functionally specialized variants [29, 90]. Notably, we and others previously observed an enrichment of genes encoding alternative proteoforms—often expressed as proteoform pairs—among proteins predisposed to form higher-order homo- or hetero-oligomeric assemblies [19], a trend recapitulated here. Our observations with, for example, *ribE* in particular motivate future-targeted studies to test assembly and stability differences among its proteoforms, as well as the potential for mixed oligomer formation when multiple proteoforms co-exist. Such targeted functional assays would provide direct evidence of how proteoform heterogeneity modulates enzyme behavior *in vivo*.

Likewise, a marked over-representation of alternative initiation events was observed among ribosomal and ribosome-associated proteins, highlighting that proteoform diversification extends to both structural and translational machineries.

Related to this, an independent analysis using PaxDB-integrated *E. coli* protein abundance estimates revealed that genes with aTISs are, on average, more highly expressed than genes with only annotated start sites, a trend that remains significant even after excluding ribosomal proteins (Supplementary Fig. S7). Together, these observations strongly suggest that expression level contributes to the detectability of aTIS events, while not precluding their occurrence in lower-abundance proteins. By providing peptide-level validation of start codon usage, TRAINSPOTTER complements Ribo-seq-based ORF discovery with confirmatory protein-level evidence. This synergy—often referred to as riboproteogenomics [29]—is vital for high-confidence genome annotation [29, 91]. Moreover, systematic validation of aTIS usage using emerging multiplexed recombineering strategies [19, 92] will be of high value for disentangling the translational and functional consequences of proteoform co-expression at single-gene resolution, and to uncover hidden layers of initiation regulation.

In sum, deformylation-assisted N-terminomics offers an orthogonal, nascent-aware complement to Ribo-seq for resolving bacterial translation initiation and mapping Nt-proteoform diversity.

## Supplementary Material

gkag587_Supplemental_Files

## Data Availability

The mass spectrometry COFRADIC proteomics data have been deposited to the ProteomeXchange Consortium via the PRIDE partner repository under the dataset identifier PXD005901 (https://dx.doi.org/10.6019/PXD005901). The datasets were generated for qualitative mapping and validation of TISs rather than for quantitative comparison between conditions; therefore, biological replicates were not included.
