# Supplementary material for: TRAINSPOTTER: profiling nascent protein N-termini indicative of bacterial translation initiation via deformylation-assisted N-terminomics
Source: Nucleic Acids Res. 2026 Jun 8;54(11):gkag587. doi: 10.1093/nar/gkag587 (PMC13245402; doi:10.1093/nar/gkag587)
Supplement: gkag587_Supplemental_Files [file gkag587_supplemental_files.zip › Supplementary Files TRAINSPOTTER.pdf]

SUPPLEMENTARY FILES

TRAINSPOTTER: Profiling Nascent Protein N-Termini Indicative of Bacterial Translation Initiation via Deformylation-Assisted N-Terminomics

Petra Van Damme[1*]

[1]RIP Unit, Laboratory of Microbiology, Department of Biochemistry and Microbiology, Faculty of Sciences, Ghent University, Ghent, Belgium

*Petra.vandamme@UGent.be

SUPPLEMENTARY FIGURES

Supplementary Figure S1

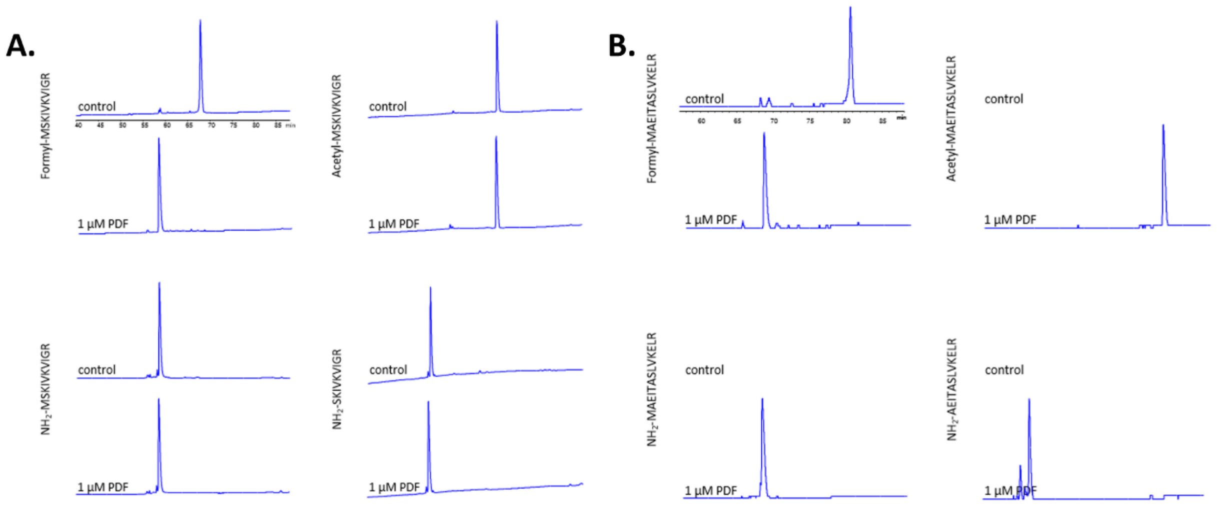

Supplementary Figure S1| Peptide deformylase (PDF) selectively deformylates Nt-formyl peptides, inducing a characteristic hydrophilic shift in RP-HPLC. Synthetic peptides representing distinct N-terminally modified variants were incubated with or without recombinant PDF (1 µM, 37°C, 1 h; see Materials and Methods) and analyzed by reversed-phase (RP)-HPLC (0.1% TFA, pH 3.0). Panels show chromatograms for two representative peptide sets: **(A)** MSKIVKVIGR variants: Nt-formyl-MSKIVKVIGR (p2499, 1157.71 Da), Nt-acetyl-MSKIVKVIGR (p2500, 1171.71 Da), Nt-free MSKIVKVIGR (p2498, 1129.71 Da), and iMet-removed Nt-free SKIVKVIGR (p2501, 998.67 Da). **(B)** MAEITASLVKELR variants: Nt-formyl-MAEITASLVKELR (p2495, 1487.8 Da), Nt-acetyl-MAEITASLVKELR (p2496, 1501.8 Da), Nt-free MAEITASLVKELR (p2494, 1459.8 Da), and iMet-removed Nt-free AEITASLVKELR (p2497, 1328.76 Da). For both sequence contexts, PDF treatment caused a pronounced and quantitative hydrophilic retention time shift (~10 min) for Nt-formylated peptides, whereas Nt-acetylated, Nt-free, and iMet-processed peptides were unaffected. Controls for non-Nt-formylated MAEITASLVKELR displayed identical RP-HPLC elution profiles before and after PDF treatment (data not shown). UV absorbance (214 nm) is shown on the Y-axis.

Supplementary Figure S2

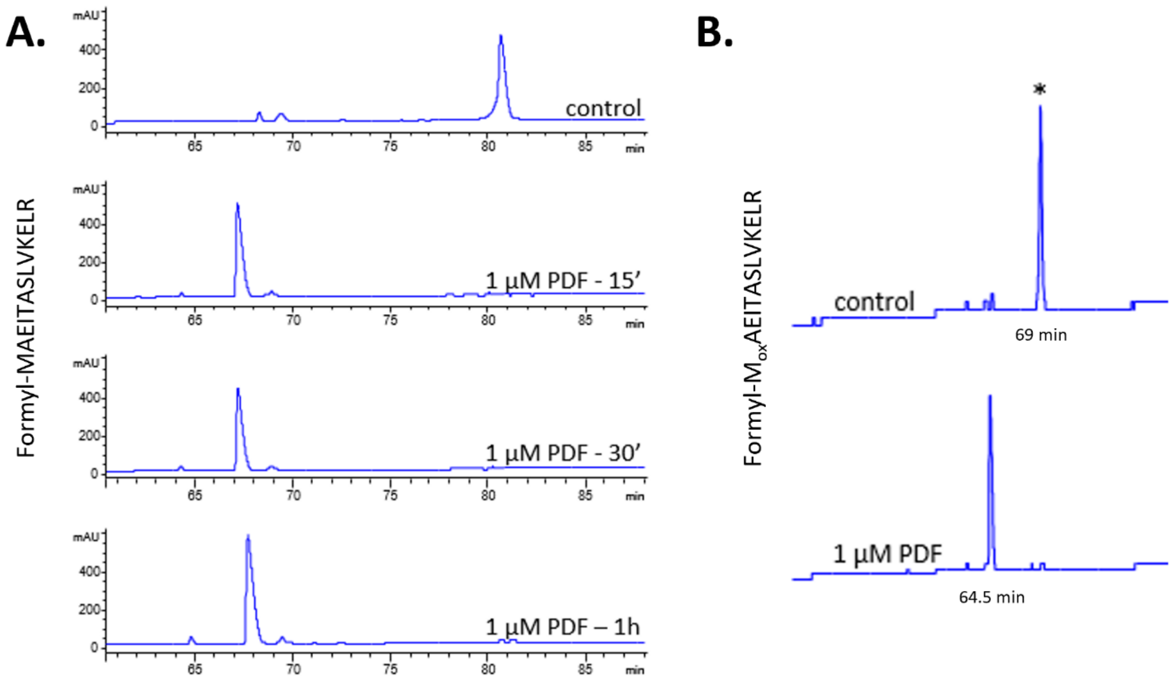

Supplementary Figure S2| Time course of peptide deformylase (PDF)-mediated deformylation and compatibility with oxidized Nt-formylmethionine. (A) RP-HPLC chromatograms showing deformylation kinetics of the Nt-formyl-MAEITASLVKELR peptide (p2495, 1487.8 Da) after incubation with recombinant PDF (1 µM) at 37°C for 15 min, 30 min, and 1 h. The control (no enzyme) shows a single major peak at ~81 min. PDF treatment quantitatively converts this peak to an earlier-eluting product (~68 min) within 15 min, with no further changes upon prolonged incubation up to 1 h. This shift is consistent with the removal of the hydrophobic Nt-formyl group and the exposure of a protonated Nt-amine under acidic conditions (0.1% TFA, pH 3.0). **(B)** RP-HPLC chromatograms of the oxidized variant, Nt-formyl-M_{ox}AEITASLVKELR ($p2495_{ox}$), before and after PDF treatment (1 µM, 37°C, 1 h). The oxidized formylated peptide elutes at ~69 min (*), and enzymatic deformylation yields a hydrophilically shifted species (~64.5 min), demonstrating that methionine oxidation does not impair PDF activity under the conditions tested. UV absorbance at 214 nm (mAU) is shown on the Y-axis.

Supplementary Figure S3

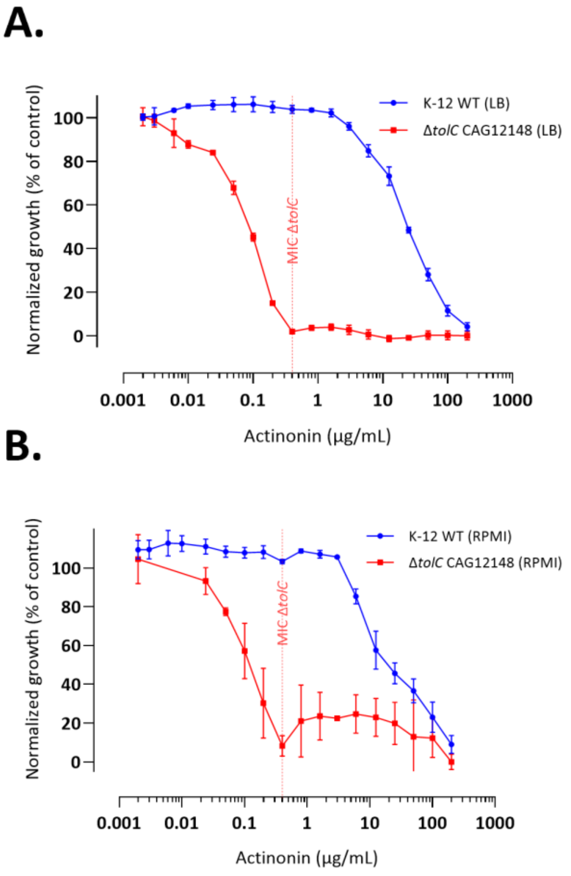

Supplemental Figure S3| Dose–response of *E. coli* K-12 WT and TolC-deficient CAG12184 to actinonin. Cultures (initial OD_{600} = 0.02; 150 µL per well) were grown overnight at 37°C with shaking (180 rpm) in 96-well plates in LB (**A**) or supplemented RPMI (**B**) and exposed to a 17-point, 2-fold serial dilution of actinonin (200–0.002 µg/mL). Growth was recorded as OD_{600} and normalized to the untreated control (mean ± SD; n = 3 per concentration). Curves show WT K-12 (blue) and CAG12184 (red). The vertical dashed line marks the MIC for CAG12184 (0.4 µg/mL in both LB and RPMI), defined as the lowest concentration with no significant growth relative to the untreated control by one-way ANOVA with Dunnett's multiple comparisons test (GraphPad Prism 10, v10.6.0; adjusted $p \leq 0.05$). The WT MIC exceeded 200 µg/mL in both media.

Supplementary Figure S4

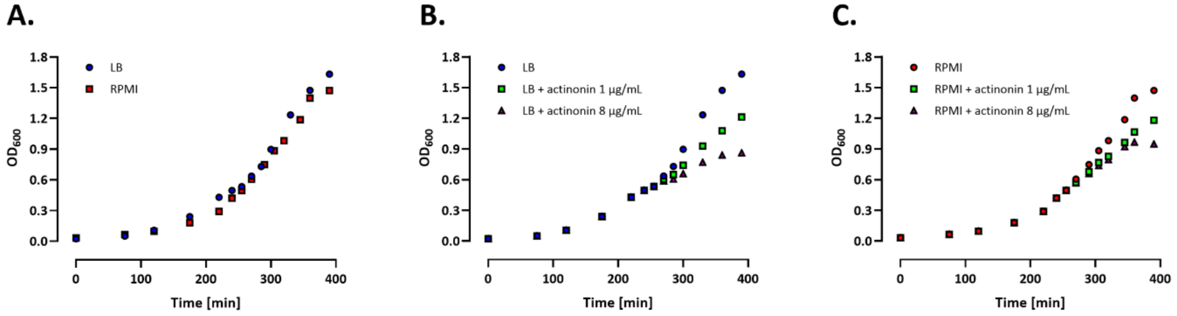

Supplemental Figure S4| Growth of *E. coli* CAG12184 in LB or SILAC-compatible RPMI medium, and effect of actinonin treatment. Optical density at 600 nm (OD_{600}) was monitored over time for *E. coli* K-12 and TolC-deficient CAG12184 cultures grown under different conditions. (**A**) Comparison of growth in rich LB medium (blue circles) and defined supplemented RPMI medium (red squares). Cultures were inoculated from overnight LB starter cultures, washed, and grown at 37°C with shaking at 180 rpm; OD_{600} was recorded at the indicated time points. Both media supported comparable exponential growth rates (LB: growth rate $\mu \approx 0.0114$ min^{-1}, doubling time $T_d \approx 60.8$ min; RPMI: $\mu \approx 0.0124$ min^{-1}, $T_d \approx 56.1$ min). (**B**) Effect of actinonin addition on growth in LB medium. Actinonin was added at $OD_{600} \approx 0.5$ (mid-log phase) to final concentrations of 1 µg/mL (green squares; post-addition $T_d \approx 97.6$ min) or 8 µg/mL (black triangles; growth almost completely arrested). Increasing actinonin concentrations led to a concentration-dependent slowdown in growth rate. (**C**) Effect of actinonin addition on growth in RPMI medium. Cultures were grown as in panel A until $OD_{600} \approx 0.5$, when actinonin was added at 1 µg/mL (green squares; post-addition $T_d \approx 96.2$ min) or 8 µg/mL (black triangles; growth almost completely arrested). Growth inhibition patterns were comparable to those observed in LB (panel B). Data are representative of at least two independent experiments. Specific growth rates (µ) were determined by fitting the natural logarithm of OD_{600} to time using linear regression within the exponential growth phase window (1) ($OD_{600} \approx 0.10$–0.50 before actinonin addition and $OD_{600} \approx$ 0.50–1.00 post-addition) using the Doubling Time Computing tool (Roth, 2006; http://www.doubling-time.com), based on the formula $T_d = \ln(2)/\mu$.

Supplementary Figure S5

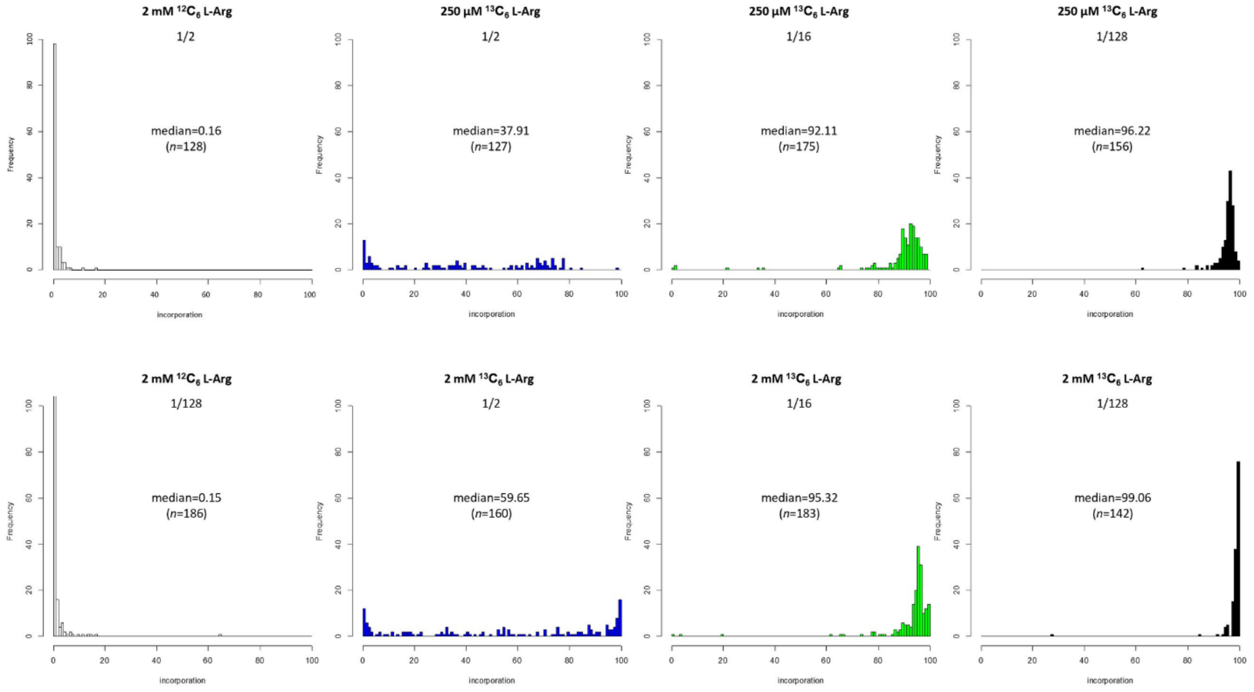

Supplemental Figure S5| Evaluation of $^{13}C_6$ L-arginine hydrochloride (Arg6) incorporation for optimized SILAC labeling in *E. coli* TolC-deficient CAG12184. *E. coli* CAG12184 cells cultured in supplemented RPMI medium were transferred to RPMI medium containing either 250 µM or 2 mM Arg6, or 2 mM $^{12}C_6$ L-arginine hydrochloride as a control, and cultured for approximately 2, 4, and 7 cell doublings. Light/heavy SILAC ratios for all identified L-Arg-containing peptide pairs were quantified from MS spectra using Mascot Distiller. Isotope incorporation efficiency was calculated as: Incorporation efficiency = (1/(ratio+1))*100. After 7 cell doublings, incorporation efficiencies approached completion with a median efficiency of 99% (*n* = 142 peptide SILAC ratios from identified peptides) for the culture supplemented with 2 mM $^{13}C_6$ L-arginine.

Supplementary Figure S6

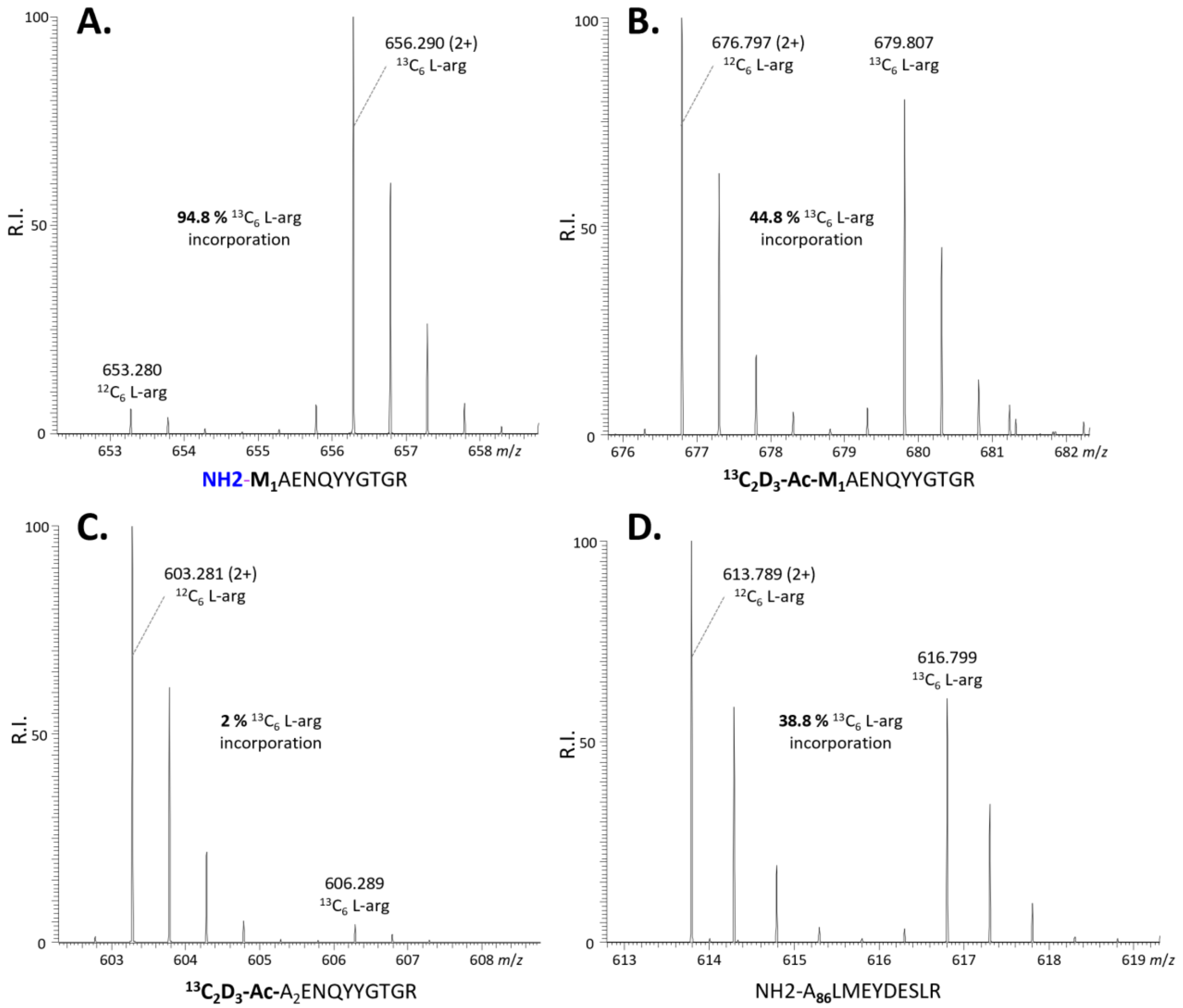

Supplemental Figure S6| Representative MS spectra of *E. coli* 30S ribosomal protein S9 peptides from distinct (N-terminal) peptide classes. Representative MS spectra are shown for peptides belonging to distinct (N-terminal) peptide classes. All peptides originate from the *E. coli* 30S ribosomal protein S9 RpsI (UniProt accession A1AGC3, gene name *rpsI*) and were identified in secondary COFRADIC fractions collected after in vitro peptide deformylation by the action of peptide deformylase (PDF). Panel A displays the Nt-free, α-amino-unmodified N-terminal peptide starting with methionine (M_1AENQYYGTGR), corresponding to a shifted-deformylated peptide generated by in vitro PDF treatment, with 94.8% $^{13}C_6$ L-arginine incorporation—indicative of high heavy-label incorporation and consistent with its nascent origin. Panel B shows the heavy Nt-

acetylated ($^{13}C_2D_3$Ac-) form of this N-terminal peptide ($^{13}C_2D_3$-Ac-M_1AENQYYGTGR), generated through in vivo deformylation followed by in vitro $^{13}C_2D_3$-Nt-acetylation, with 44.8% incorporation. Panel C shows the heavy Nt-acetylated ($^{13}C_2D_3$-Ac-) and iMet-processed variant ($^{13}C_2D_3$-Ac-A_2ENQYYGTGR), with only 2% incorporation. This low labeling level matches the Nt-heavy-acetylated ($^{13}C_2D_3$-Ac-) peptide population in **Figure 2A** (median = 1.5%, mean = 24%) and is frequently indicative of proteolytic N-terminal proteoforms—here, generated by co- or post-translational iMet removal. Panel D presents an internal tryptic ArgC/P peptide (A_{86}LMEYDESLR), with 38.8% incorporation, in line with Nt-free, α-amino–unmodified peptides in **Figure 2A** (median = 37%, mean = 41%). All peptides were derived from cultures grown in RPMI medium to an $OD_{600} \approx 0.5$, supplemented with 2 mM $^{13}C_6$ L-arginine hydrochloride and 1 µg/mL actinonin, and incubated for an additional 1 h (final $OD_{600} \approx 0.85$) prior to harvesting. Together, these data illustrate the substantially higher contribution of recent translation to the in vivo formylated (and in vitro PDF-deformylated) N-terminal peptide (panel A) compared with both the internal peptide (panel D) and its in vivo deformylated (and in vitro $^{13}C_2D_3$-Nt-acetylated) counterpart (panel B). The N-terminal peptide corresponding to the Nt-heavy-acetylated ($^{13}C_2D_3$-Ac-) and iMet-processed proteolytic N-terminal 30S ribosomal protein S9 proteoform in panel C predominantly represents a pool derived from earlier co- or post-translation events, with only a minor fraction reflecting recent synthesis.

Supplementary Figure S7

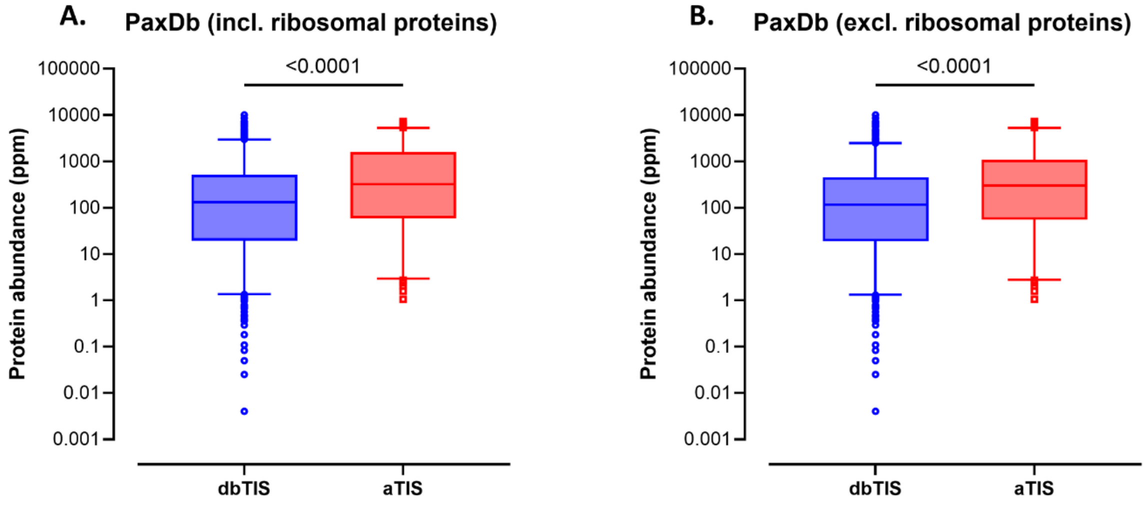

Figure S7| Protein abundance distributions of genes with alternative versus annotated translation initiation sites. PaxDB-integrated protein abundance estimates for *Escherichia coli* K-12 MG1655 (PaxDB v6.0; whole-organism integrated dataset) were used to compare genes with database-annotated translation initiation sites (dbTISs; 687 unique protein matches—MgtL [b4702] lacked a corresponding PaxDB entry) and genes harboring at least one alternative translation initiation site (aTISs; 107 unique protein matches) identified by TRAINSPOTTER. **(A)** Boxplot showing log_{10}-transformed protein abundance distributions including ribosomal proteins. **(B)** Boxplot showing log_{10}-transformed protein abundance distributions, after exclusion of ribosomal proteins. Boxes indicate the median and interquartile range; whiskers denote the 5th–95th percentiles. Statistical significance was assessed using a two-sided Wilcoxon rank-sum test. Genes harboring aTISs display significantly higher protein abundance than dbTIS-associated genes both when all proteins are considered (median log_{10} abundance 2.51 vs 2.12; $p = 1.62 \times 10^{-5}$) and after exclusion of ribosomal proteins (median 2.48 vs 2.06; $p = 5.37 \times 10^{-5}$).

SUPPLEMENTARTY TABLE LEGENDS

Supplementary Table S1| Comprehensive list of unique *E. coli* N-termini and corresponding translation initiation sites (TISs) identified by TRAINSPOTTER. This table summarizes all unique protein N-terminal peptides (1082) identified in this study. Each entry represents a distinct N-terminus mapped to a genomic locus, grouped by sequence identity and annotated with the corresponding *E. coli* gene(s), locus tag, UniProt accession, and proteoform information. Distinct N-terminal modification states (e.g., formylated, free, acetylated, heavy-acetylated) are listed separately per sequence to illustrate the diversity of co-existing Nt-proteoforms detected for a given start site. For each N-terminus, the table integrates proteomic and ribosome profiling evidence. Columns *Ribo-seq evidence*, *Ribo-RET evidence*, and *Ribo-seq elongation evidence* indicate independent support from previously published ribosome-profiling analyses of *E. coli* translation initiation or elongation (2-5). The column *TIS category* specifies whether the N-terminus corresponds to a database-annotated translation initiation site (dbTIS) or an alternative initiation site (aTIS), either upstream or downstream and in-frame relative to the canonical start. The fields *Start codon*, *Start codon type*, and *dbTIS start codon* define the initiating triplet and classify it as cognate (ATG), near-cognate (ATC, TTG, GTG, ATT, ACG, AAG, CTG), or non-cognate, together with strand orientation and precise genomic coordinates. *Gene names*, *Accession ProteoMapper*, *Entry name*, and *Protein names* provide UniProt-based identifiers and standard nomenclature. *Start incl. extra iMet UniProt*, *End UniProt*, *Length UniProt (aa)*, *Length proteoform (aa)*, and *Length difference* report positional offsets and size variation between annotated and experimentally detected proteoforms, while *Proteoform info* summarizes N-terminal sequence features, iMet processing, and chemical modification states. Quantitative columns detail peptide-level MS/MS-evidence for translation initiation. *Acetyl (iMet compatible N-term)*, *Formyl iMet (N-term)*, *Heavy acetyl (N-term)*, *Oxidation M/unique peptide*, *NH2-M/unique peptide*, and *NH2-M shifted evidence (combo fr 1–22)* indicate the specific N-terminal modifications observed and whether *NH2-M* peptides were detected in the deformylation-shifted (TRAINSPOTTER) fractions. The column *Bienvenut et al. (Nt-formyl-supported dbTIS)* indicates overlap with Nt-formylation-supported annotated initiation sites reported by Bienvenut et al. (6). *First AA of sequence* and *Sequence (incl. extra M(ox))* show the experimentally verified peptide sequence,

and SILAC-derived ratios (*Nt-free avg SILAC H/L*, *Nt-HeavyAc avg SILAC H/L*, *Nt-Ac avg SILAC H/L*, and *Formyl avg SILAC TL H/L*) distinguish nascent from pre-existing proteoforms in the total lysate (TL), confirming translation activity at initiation sites. The genomic context of each site is provided through *dbTIS start/stop/coordinates*, *CDS coordinates (GCF)*, *Annotated CDS stop*, *Inferred TIS start/stop/coordinates*, and *Inferred CDS coordinates*, allowing direct comparison between annotated and experimentally inferred start sites. *Inferred TIS ±3 codons* indicates proximity to the annotated site, while *Context_60nt*, *Frame*, and *Proxy SD (AGG, GGA)* with *Best spacing nt* describe the 60-nt context surrounding the inferred start, the reading-frame assignment, and predicted Shine–Dalgarno-like motifs with effective ribosome-binding-site spacing (3–15 nt upstream). *Window −30..+15 RNA*, *mfe dbTIS*, and *mfe aTIS* provide local RNA sequence windows and the corresponding minimum free-energy (MFE) values used to evaluate predicted mRNA secondary-structure stability near initiation sites (see Materials and Methods). Protein-level annotations include predicted targeting and (UniProt) topology information such as *SignalP-6.0*, *Signal peptide*, and *Transmembrane*. Functional annotation is summarized under *Domain [CC]*, *Sequence (annotated proteoform)*, *Function [CC]*, *Keywords*, and *Gene Ontology* (biological process, cellular component, molecular function). *Mass (annotated proteoform)* lists the theoretical molecular mass of the corresponding UniProt reference sequence.

Supplementary Table S2 | 68 genes with multiple translation initiation sites (TISs) evidenced by distinct N-terminal peptides. This table, derived from **Table S1**, summarizes at the gene level all loci for which more than one in-frame TIS is supported by unique N-termini in the TRAINSPOTTER dataset. Each row corresponds to a single locus and lists every in-frame TIS supported by N-terminal peptide evidence. *Locus_tag* and *Gene_names* report the chromosomal locus tag and the associated gene name(s). *Strand* indicates gene orientation (“+” or “−”); all ordering and the definition of “upstream/downstream” follow the 5′ to 3′ direction of the coding strand (for “−” strand genes, upstream has the larger genomic coordinate). *CDS_stop* gives the genomic coordinate of the annotated stop codon shared by all TISs for the locus. *n_TIS* is the number of distinct in-frame TIS supported by unique N-termini for that locus. *dbTIS_included* indicates whether the database-annotated TIS (dbTIS) is supported by N-terminal peptide evidence in this dataset (“yes” or “no”). Offsets in this table are expressed in codons relative to

the most 5′ (upstream) identified TIS (*Offset reference* with uTIS (upstream TIS relative to the annotated start), dbTIS (the database-annotated TIS), or dTIS (downstream TIS relative to the annotated start)) on the coding strand. *dbTIS_offset_from_upstream_TIS* reports the position of the dbTIS relative to that upstream reference ("0" if the upstream TIS coincides with the dbTIS; a positive value if the dbTIS lies downstream); when *dbTIS_included* is "no", this value is inferred from the annotation to indicate the expected placement of the dbTIS with respect to the observed TIS(s). *Start_coordinates_5′_to_3′* lists the genomic start coordinates of all supported TIS ordered 5′ to 3′ on the coding strand. *Offsets_from_upstream_TIS* lists the codon offsets of all alternative TIS relative to the upstream TIS (the upstream reference itself is "0" by definition and is not repeated). *TIS_evidence* provides, in upstream to downstream order, a compact summary for each TIS in the form "offset [StartCodon]: peptide(s) (start–end)", where the offset is defined as above, the bracketed label denotes the consensus start codon assigned to that TIS, and the peptide list contains the unique N-terminal peptide sequence(s) with their UniProt residue ranges in parentheses.

References

1. Monod, J. (1949) The growth of bacterial cultures. *Annu Rev Microbiol*, **3**, 371-394.
2. Meydan, S., Marks, J., Klepacki, D., Sharma, V., Baranov, P.V., Firth, A.E., Margus, T., Kefi, A., Vazquez-Laslop, N. and Mankin, A.S. (2019) Retapamulin-Assisted Ribosome Profiling Reveals the Alternative Bacterial Proteome. *Mol Cell*, **74**, 481-493 e486.
3. Hor, J., Jung, J., Ethurica-Mitic, S., Barquist, L. and Vogel, J. (2022) INRI-seq enables global cell-free analysis of translation initiation and off-target effects of antisense inhibitors. *Nucleic Acids Res*, **50**, e128.
4. Ndah, E., Jonckheere, V., Giess, A., Valen, E., Menschaert, G. and Van Damme, P. (2017) REPARATION: ribosome profiling assisted (re-)annotation of bacterial genomes. *Nucleic Acids Res*, **45**, e168.
5. Stringer, A., Smith, C., Mangano, K. and Wade, J.T. (2021) Identification of novel translated small ORFs in Escherichia coli using complementary ribosome profiling approaches. *J Bacteriol*, **204**, JB0035221.
6. Bienvenut, W.V., Giglione, C. and Meinnel, T. (2015) Proteome-wide analysis of the amino terminal status of Escherichia coli proteins at the steady-state and upon deformylation inhibition. *Proteomics*, **15**, 2503-2518.
